# Diagnosis of colonic dysmotility associated with autonomic dysfunction in patients with chronic refractory constipation

**DOI:** 10.1038/s41598-022-15945-6

**Published:** 2022-07-14

**Authors:** Lijun Liu, Natalija Milkova, Sharjana Nirmalathasan, M. Khawar Ali, Kartik Sharma, Jan D. Huizinga, Ji-Hong Chen

**Affiliations:** Division of Gastroenterology, Department of Medicine, Farncombe Family Digestive Health Research Institute, McMaster University, HSC-3N8E, 1200 Main Street West, Hamilton, ON L8N 3Z5 Canada

**Keywords:** Functional gastrointestinal disorders, Motility disorders, Biomarkers, Gastroenterology, Medical research

## Abstract

We report the first study assessing human colon manometric features and their correlations with changes in autonomic functioning in patients with refractory chronic constipation prior to consideration of surgical intervention. High-resolution colonic manometry (HRCM) with simultaneous heart rate variability (HRV) was performed in 14 patients, and the resulting features were compared to healthy subjects. Patients were categorized into three groups that had normal, weak, or no high amplitude propagating pressure waves (HAPWs) to any intervention. We found mild vagal pathway impairment presented as lower HAPW amplitude in the proximal colon in response to proximal colon balloon distention. Left colon dysmotility was observed in 71% of patients, with features of (1) less left colon HAPWs, (2) lower left colon HAPW amplitudes (69.8 vs 102.3 mmHg), (3) impaired coloanal coordination, (4) left colon hypertonicity in patients with coccyx injury. Patients showed the following autonomic dysfunction: (1) high sympathetic tone at baseline, (2) high sympathetic reactivity to active standing and meal, (3) correlation of low parasympathetic reactivity to the meal with absence of the coloanal reflex, (4) lower parasympathetic and higher sympathetic activity during occurrence of HAPWs. In conclusion, left colon dysmotility and high sympathetic tone and reactivity, more so than vagal pathway impairment, play important roles in refractory chronic constipation and suggests sacral neuromodulation as a possible treatment.

## Introduction

The colon remains a poorly understood organ, although chronic constipation has a significant impact on patients’ quality of life and is a major public health issue worldwide, both in the pediatric and adult populations^1^. Chronic constipation is challenging to treat and is debilitating^2,3^. A better understanding of the pathophysiology of colonic dysfunction is needed^4^. Colonic motility testing is necessary to identify if constipation is caused by colonic motor dysfunction, but it is rarely done in adults. Hence treatment of severe refractory constipation is done empirically. Surgery, which is often performed without physiological testing, is still an option but is associated with significant morbidity and does not decrease resource utilization^5,6^.

Human colonic transit and defecation are neuronally regulated through multi-level neuronal reflexes, such as the gastrocolic reflex (e.g. a colonic motor response to gastric distention) and the sacral defecation reflex (evoking propulsive activity in the left colon by rectal bisacodyl). A vagal autonomic reflex can be initiated by a meal, proximal colon distention or rectal bisacodyl stimulation to evoke propulsive activity starting from the proximal colon. The coloanal reflex involves the relaxation of the anal sphincters in response to a propulsive colon motor activity. Functional or structural deficits in the neural circuits may lead to colonic dysfunction due to abnormal or absent reflexes. Sensory information from the musculature and lumen of the colon, rectum and anal canal^7,8^ is sent to the spinal cord, including the sacral parasympathetic nucleus^9^ and up into the brainstem^10^ including the nucleus tractus solitarius (NTS)^11^. The sensory information is integrated in the brainstem, influenced by the cortex, which can lead to stimulation of excitatory vagal and spinal parasympathetic motor neurons to initiate motor patterns in the proximal, transverse or descending colon^7,10^. The importance of the extrinsic autonomic nervous system (ANS) in the regulation of defecation reflexes has been demonstrated in animal models^12^ and in patients with spinal injury who lost spontaneous bowel movements due to damage to sacral parasympathetic innervation^13,14^.

Despite our extensive knowledge of the extrinsic innervation of the colon, it is largely ignored in the diagnosis of functional colonic disorders. A symptom-based diagnosis of constipation evaluates only transit or features of obstructive defecation and provides limited understanding of individual pathophysiology phenotypes, insufficient for effective management.

High-resolution colonic manometry (HRCM) allows for a detailed assessment of pressure changes throughout the colon, including biomarkers such as high-amplitude propagating pressure waves (HAPWs) and simultaneous pressure waves (SPWs). During HRCM using water-perfused catheters, both motor patterns are associated with liquid and gas expulsion^15,16^. The present study emphasized HAPWs since this is the motor pattern most associated with transit, with the preparatory phase of defecation^17^ and with the act of defecation^18,19^. The objective to evaluate the ability of patients to generate HAPW activity precluded a protocol that only examines baseline and meal response; in healthy subjects, these conditions do not always evoke HAPWs^20^. We included proximal colon balloon distention (PBD) and rectal bisacodyl administration since these were optimal stimuli to evoke HAPWs in healthy subjects^20^. We subscribe to the conclusion by Bassotti et al. that observation of normal stimulus-evoked responses will encourage further therapeutic efforts^21^. The HAPWs evoked by balloon distention or bisacodyl do not differ in characteristics from those occurring under physiological conditions^20,21^ since they evoke natural reflex motor patterns. PBD evokes a vagally mediated reflex and rectal bisacodyl can evoke both a sacral and a vagal reflex, with HAPWs propagating from the proximal to the left colon and coordinated anal sphincter relaxation. Patients with constipation may show a variety of reflex impairments.

Activities of the sympathetic and parasympathetic branches of the ANS have been measured through heart rate variability (HRV)^22–24^. Our previous studies have shown a correlation between HRV parameters and colonic motor patterns in healthy volunteers, whereby HAPWs were associated with an increase in parasympathetic activity and a decrease in sympathetic activity^15,25^. Among the multiple HRV parameters we have previously tested, respiratory sinus arrhythmia (RSA) and the sympathetic index (SI), also known as Baevsky’s stress index, were the best parameters for observing the parasympathetic and sympathetic nervous system activity related to both the active standing test and the HAPW.

The objective of our study was to assess the pathophysiology of chronic refractory constipation by analysis of colonic motor patterns and colonic autonomic reflexes using HRCM, and determine whether autonomic dysfunction was associated with colonic dysmotility to provide a guide for optimal personalized management. The underlying hypothesis was that inhibition of HAPW activity is due to high sympathetic activity and/or low parasympathetic activity.

We also aimed to discover essential biomarkers of pathophysiology, and relationships between spinal injury, colonic dysmotility and autonomic dysfunction. We hypothesized the involvement of interstitial cells of Cajal (ICC) in colonic dysmotility through a detailed frequency analysis of all rhythmic pressure waves. The patient data were compared with those of a cohort of healthy subjects without gastrointestinal or autonomic or cardiac disorders that underwent the same interventions under identical conditions^15,16,20^.

## Results

All patients were diagnosed according to the Rome IV criteria for chronic constipation, characterized by long-standing constipation, poor response to medication, and insufficiency of spontaneous bowel movements requiring a combination of laxatives, stool softeners, and enemas (Supplementary Table S1).

### Characteristics of HAPWs

**Figure 1:**
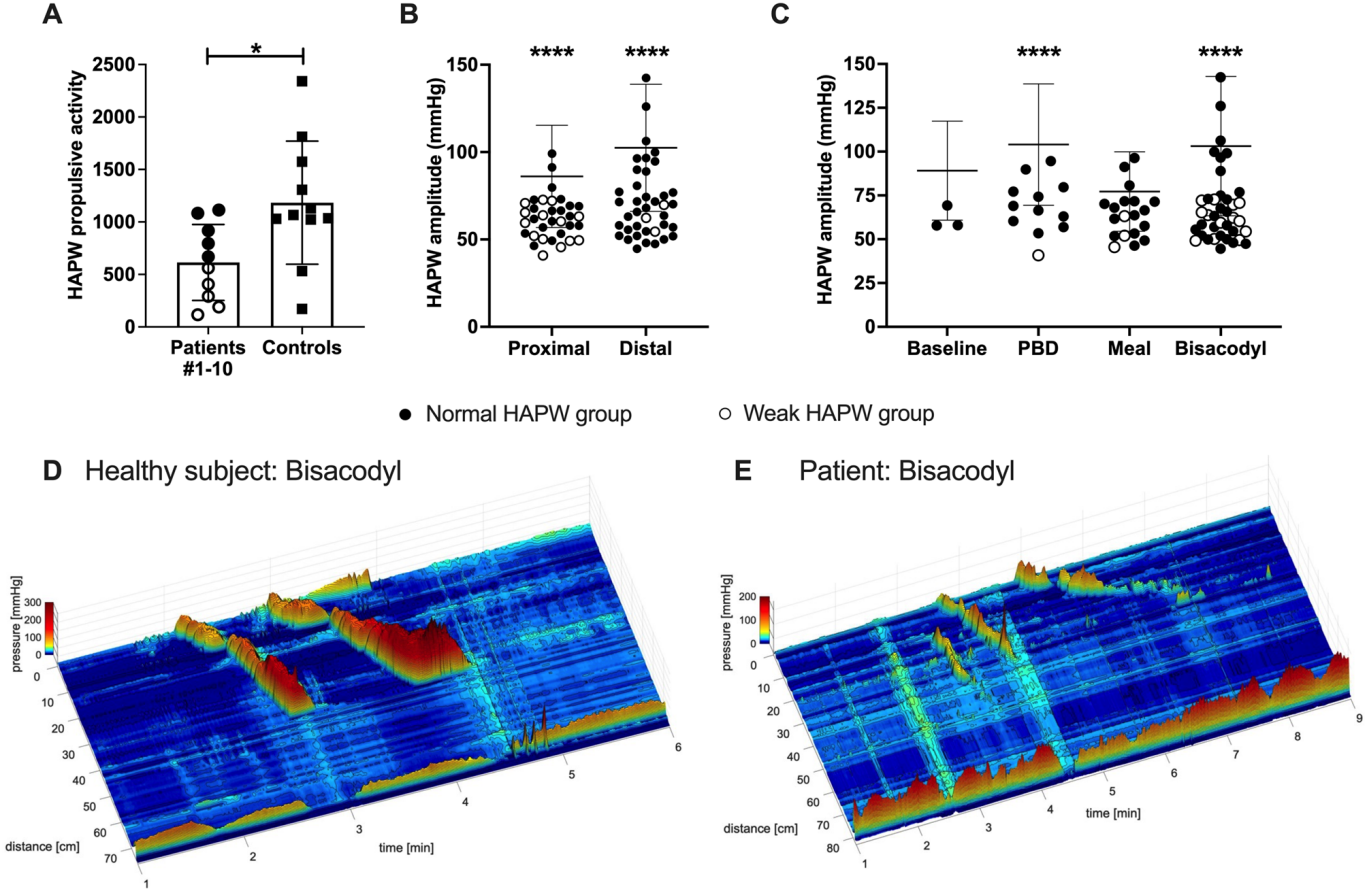
The amplitude of HAPWs and the HAPW Propulsive Activity was lower in patients compared to that of healthy controls^20^. (**A**) Propulsive activity is defined as the average amplitude of HAPWs x the number of HAPWs present during the entire protocol. Patients (N = 10) were separated into a weak and a normal HAPW group based on whether their HAPW propulsive activity was within one standard deviation of the healthy control (N = 11) mean value. Overall, patients had significantly lower HAPW propulsive activity than healthy subjects. Patients without HAPWs (Patients #11–14) were excluded. * P < 0.05. (**B**) Amplitude of HAPWs that started in the proximal and the distal colon (left colon). Patients (N = 14, n = 73) showed significantly lower proximal and distal colon HAPW amplitude than healthy subjects(N = 19, n = 185). Lines show mean ± SD of HAPWs in healthy subjects. (**C**) HAPW amplitude in patients during baseline, in response to proximal balloon distention (PBD), in response to a 1000 kcal meal, and rectal bisacodyl administration. Patients (N = 14, n = 73) showed significantly lower HAPW amplitude than healthy subjects (N = 19, n = 185) in response to PBD and rectal bisacodyl. Lines show mean ± SD of HAPWs in healthy subjects. **** P < 0.0001. (**D**) Typical map of HAPWs observed in healthy subjects. The HAPWs that propagated from the proximal ascending colon (at 0 cm) and terminated in the descending colon (at 38 cm) were evoked by rectal bisacodyl. The anal sphincter (at 72 cm) relaxed in association with each HAPW. (**E**) Typical map of HAPWs observed in a patient. HAPWs were of lower amplitude compared to healthy subjects. These HAPWs stimulated by rectal bisacodyl in patient #2 propagated from the proximal descending colon (at 0 cm) to the sigmoid colon (at 33 cm). Only one HAPW was associated with anal sphincter (at 78 cm) relaxation.

A total of 73 propagating pressure waves (including HAPWs and LAPWs) were observed in the 14 patients, providing an average of 5 per patient. In contrast, a total of 185 HAPWs were observed in 19 healthy subjects in response to the same interventions, providing an average of 10 HAPWs per healthy subject^20^. Only 2 (14%) patients had spontaneous HAPW activity during baseline, compared to 8 of 19 (42%) in healthy subjects (Tables 1, 2). The HAPW “propulsive activity” (average amplitude × number of HAPWs (see “Methods”)) of patients with HAPWs was significantly lower than that of healthy subjects (P = 0.0151) (Fig. 1A). Patients showed variability in HAPW activity, including normal HAPW activity (patients #1–5), weak HAPW activity (patients #6–10) where at least one HAPW was evoked but with HAPW propulsive activity below 1 SD of the mean of healthy subjects, and patients who had no HAPW activity (patients #11–14) (Table 1, Fig. 1). The amplitudes of HAPWs were significantly lower in patients in comparison to healthy subjects (Fig. 1B,C, Table 1).

**Table 1:**
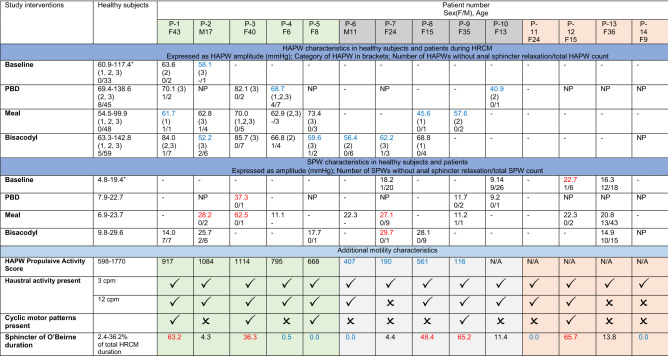
Motility characteristics of all patients in comparison with healthy subjects.

*HAPW* high-amplitude propagating pressure waves, *SPW* simultaneous pressure waves, *HRCM* high-resolution colonic manometry.

NP indicates intervention not performed, “−” indicates no appearance.

^+^Healthy control range of HAPW and SPW amplitude = mean ± 1SD; derived from previous studies^16, 20^.

Control SPW amplitude for bisacodyl includes both proximal colon and rectal administrations.

HAPW categories: 1 = HAPW that originates in the ascending colon 2 = HAPW that starts in the ascending colon and terminates in the transverse, or descending, or sigmoid colon, or may reach the rectum 3 = HAPW that originates at the transverse or descending colon^20^.

HAPW propulsive activity of healthy subjects (N = 11) were derived from healthy subjects who received the same interventions^20^.

Normal anal relaxation was defined as relaxation of > 30% in anal sphincter pressure for HAPWs, and > 25% for SPWs^16, 20^.

Blue font = value is lower than 1 standard deviation of healthy control mean.

Red font = value is higher than 1 standard deviation of healthy control mean.

**Table 2:** Proportion of HAPW categories, occurrence of HAPW response to interventions, and autonomic reflexes during HRCM in chronic constipation patients and healthy subjects.

	Patients (n/N (%))	Healthy control (n/N (%))	P value
	**Proportion of HAPW categories**		
Proximal HAPWs	12/73 (16%)	62/290 (21%)	0.3490
Full HAPWs	20/73 (27%)	85/290 (29%)	0.7473
Descending HAPWs	41/73 (56%)	143/290 (49%)	0.2951
	**Presence of evident functional autonomic pathways**		
Vagal pathway	10/14 (71%)	18/19 (95%)	0.1376
Sacral pathway	7/14 (50%)	17/19 (89%)	0.0191*
	**Presence of HAPW response to individual interventions of HRCM**		
Spontaneous HAPWs	2/14 (14%)	8/19 (42%)	0.1312
Proximal balloon distention	4/8 (50%)	16/19 (84%)	0.1445
Rectal bisacodyl	8/13 (62%)	12/13 (92%)	0.1602
Meal	7/14 (50%)	13/19 (68%)	0.4720
	**Presence of HAPWs with ascending colon origin**		
Proximal balloon distention	2/8 (25%)	4/19 (21%)	1.0000
Rectal bisacodyl	4/8 (50%)	8/13 (62%)	0.6731
Meal	5/8 (62%)	9/19 (47%)	0.6776
	**Presence of transverse and descending originating HAPWs**		
Proximal balloon distention	3/8 (38%)	16/19 (84%)	0.0267*
Rectal bisacodyl	5/13 (38%)	8/13 (62%)	0.4338
Meal	4/14 (29%)	8/19 (42%)	0.4861
	**Presence of autonomic reflex during HRCM**		
Coloanal reflex	7/12 (58%)	19/19 (100%)	0.0047**
Gastrocolic reflex	8/14 (57%)	15/19 (79%)	0.2569
Sacral autonomic reflex	5/13 (38%)	11/16 (69%)	0.1436
Vagosacral reflex	4/8 (50%)	13/19 (68%)	0.4147

Total number of subjects = N, number of subjects with response = n. P values are reported for the Chi-squared test for the proportion of HAPW categories and the Fischer exact test for the presence of HAPW and autonomic reflex response compared to proportions. The proportion of HAPW category and response data of healthy controls were derived from Milkova et al.^20^. HAPW categories, the number of SPWs, and anal sphincter relaxation were subsequently used to determine the presence of reflexes. *P ≤ 0.05; **P ≤ 0.01.

### Generation of HAPWs via the vagal pathway

Vagal sensory nerves, when stimulated by a 1000 kcal meal or PBD, may lead to a motor response in the proximal and left colon with the involvement of vagal and sacral efferent pathways.

PBD evoked HAPWs in 50% of the patients and in 84% of healthy subjects (Tables 1, 2). Weakness of the vagal pathway in the patients’ HAPW response was also shown by a significantly lower amplitude of HAPWs evoked by PBD in patients compared to healthy subjects (Fig. 1C) (P < 0.0001). A meal evoked HAPWs in 50% of patients compared to 68% of healthy subjects, but the average HAPW amplitude was not statistically significantly different (65 mmHg vs. 77 mmHg, P = 0.0518, Fig. 1C). The meal-evoked gastrocolic reflex, defined as an increase in the generation of HAPWs or SPWs in the 90 min following meal intake compared to baseline, was present in 8 of 14 (57%) patients compared to 15 of 19 (79%) healthy subjects (Tables 1, 2).

The vagal efferent pathway can also be stimulated by rectal bisacodyl via spinal afferent pathways. Taken together, PBD, meal intake, and rectal bisacodyl generated proximal colon HAPWs and/or the gastrocolic reflex in 10 of 14 (71%) patients compared to 18 of 19 (95%) healthy subjects (Table 2). The HAPW amplitudes were significantly lower in patients than in healthy subjects (Fig. 1B,C).

Vagal pathway activity, including the vagosacral reflex and the gastrocolic reflex, was observed in most patients from both the normal and weak HAPW groups, where it was only absent in patient #7. In contrast, 3 of the 4 patients without HAPWs did not show any evidence of vagal pathway activity (Table 3, Patients #11, 12, 14).

**Table 3:** Presence of autonomic reflexes, high sphincter of O’Beirne activity, and autonomic dysfunction in individual patients.

F/M Age	Autonomic reflexes				High sphincter of O’Beirne activity	High sympathetic activity, low parasympathetic activity and/or high SI/RSA			Pathophysiology hypothesis
	Vagosacral reflex	Gastrocolic reflex	Sacral autonomic reflex	Coloanal reflex		Supine tone	Active standing test	Colonic stimuli	
**Normal HAPW group**									
P-1 F43	✓	×	✓	×	✓	×	✓	✓	Dominant sympathetic reactivity to standing and colonic stimuli may inhibit the gastrocolic reflex and contribute to coloanal dyssynergia and high sphincter of O’Beirne activity
P-2 M17	ND	✓	✓	×	×	×	×	✓	Dominant sympathetic reactivity to colonic stimuli may contribute to coloanal dyssynergia
P-3 F40	✓	✓	✓	✓	✓	×	×	✓	Dominant sympathetic reactivity to colonic stimuli may contribute to high sphincter of O’Beirne activity
P-4 F6	✓	✓	×	×	×	✓	✓	✓	Dominant sympathetic tone and reactivity may contribute to coloanal dyssynergia and absence of sacral autonomic reflex
P-5 F8	ND	✓	✓	✓	×	✓	✓	✓	Dominant sympathetic tone and reactivity may reduce colonic motility
**Weak HAPW group**									
P-6 M11	✓	✓	×	✓	×	×	×	✓	Dominant sympathetic reactivity only to rectal bisacodyl may contribute to the absence of sacral autonomic reflex
P-7 F24 Figure 5A–E	ND	×	✓	✓	×	✓	✓	✓	Dominant sympathetic tone and reactivity may contribute to the absence of the gastrocolic reflex
P-8 F15	×	✓	×	✓	✓	✓	✓	×	Dominant sympathetic tone and reactivity to standing may contribute to weak HAPW activity and high sphincter of O’Beirne activity, but not the absence of vagosacral and sacral autonomic reflex which were associated with high parasympathetic reactivity to stimuli
P-9 F35	×	✓	×	✓	✓	×	×	×	High sphincter of O’Beirne activity and the absence of vagosacral and sacral autonomic reflex were not due to dominance of sympathetic activity
P-10 F13	×	×	×	×	×	✓	✓	✓	Dominant sympathetic tone and reactivity may contribute to coloanal dyssynergia and the absence of multiple autonomic reflexes
**No HAPW group**									
P-11 F24	×	×	×	ND	×	×	✓	✓	Dominant sympathetic reactivity may contribute to the absence of multiple autonomic reflexes
P-12 F15 Figure 5F–J	ND	×	×	✓	✓	×	×	✓	Dominant sympathetic reactivity may contribute to the absence of gastrocolic and sacral autonomic reflex, and high sphincter of O’Beirne activity
P-13 F36	ND	✓	×	×	×	✓	✓	✓	Dominant sympathetic tone and reactivity may contribute to coloanal dyssynergia and the absence of sacral autonomic reflex
P-14 F9	ND	×	ND	ND	×	✓	✓	✓	Dominant sympathetic tone and reactivity may contribute to the absence of gastrocolic reflex

ND: Not determined due to catheter position for vagosacral reflex, or lack of HAPWs and SPWs for coloanal reflex.

Vagosacral reflex = proximal colon originating HAPW or LAPW that travels into the left colon, it includes category 2 HAPWs (Table 1) only if it travels into the left colon, and is evoked with or without an external stimulus.

Gastrocolic reflex = response to a meal by an increase in HAPWs and/or SPWs from baseline.

Sacral autonomic reflex = Distal colon originating HAPW in response to rectal bisacodyl.

Coloanal reflex = Anal sphincter relaxed by more than 30% when associated with HAPW and LAPWs, or more than 25% when associated with SPWs. Failed relaxation associated with more than one HAPW in one intervention, one or more HAPWs in all interventions, or more than 33% of SPWs when fewer than two HAPWs are present indicates coloanal dyssynergia.

Patients with normal HAPWs have HAPW propulsive activity score within one standard deviation from healthy subject mean value. Weak HAPW group indicate the propulsive activity score that is less than one standard deviation below the mean. No HAPW group did not have any HAPWs.

High or low HRV parameters in patients were defined as values greater or less than 1SD from the mean of healthy subjects, details are reported in Supplementary Table S5.

### Left colon motility stimulated via the sacral spinal pathway

The sacral sensory pathway function was assessed by the patients’ response to rectal bisacodyl. The sacral motor pathway function was assessed by evaluating the occurrence of HAPW activity in the left colon. Overall, sacral pathway activity was shown in 7 of the 14 (50%) patients, which was significantly less than 17 of 19 (89%) healthy subjects (P = 0.0191) (Table 2).

Transverse-descending colon originating HAPWs with activity mainly in the descending colon indicated left colon motor function, which was significantly lower in amplitude in patients compared to healthy subjects (69.8 mmHg vs. 102.2 mmHg, P < 0.0001, Fig. 1B). PBD evoked transverse-descending HAPWs in a significantly lower percentage of patients compared to healthy subjects (38% vs. 84%, P = 0.0267, Table 2). The intake of the meal elicited descending colon HAPWs in 4 of 14 (29%) patients, compared to 8 of 18 (42%) healthy subjects (Tables 1, 2).

**Figure 2:**
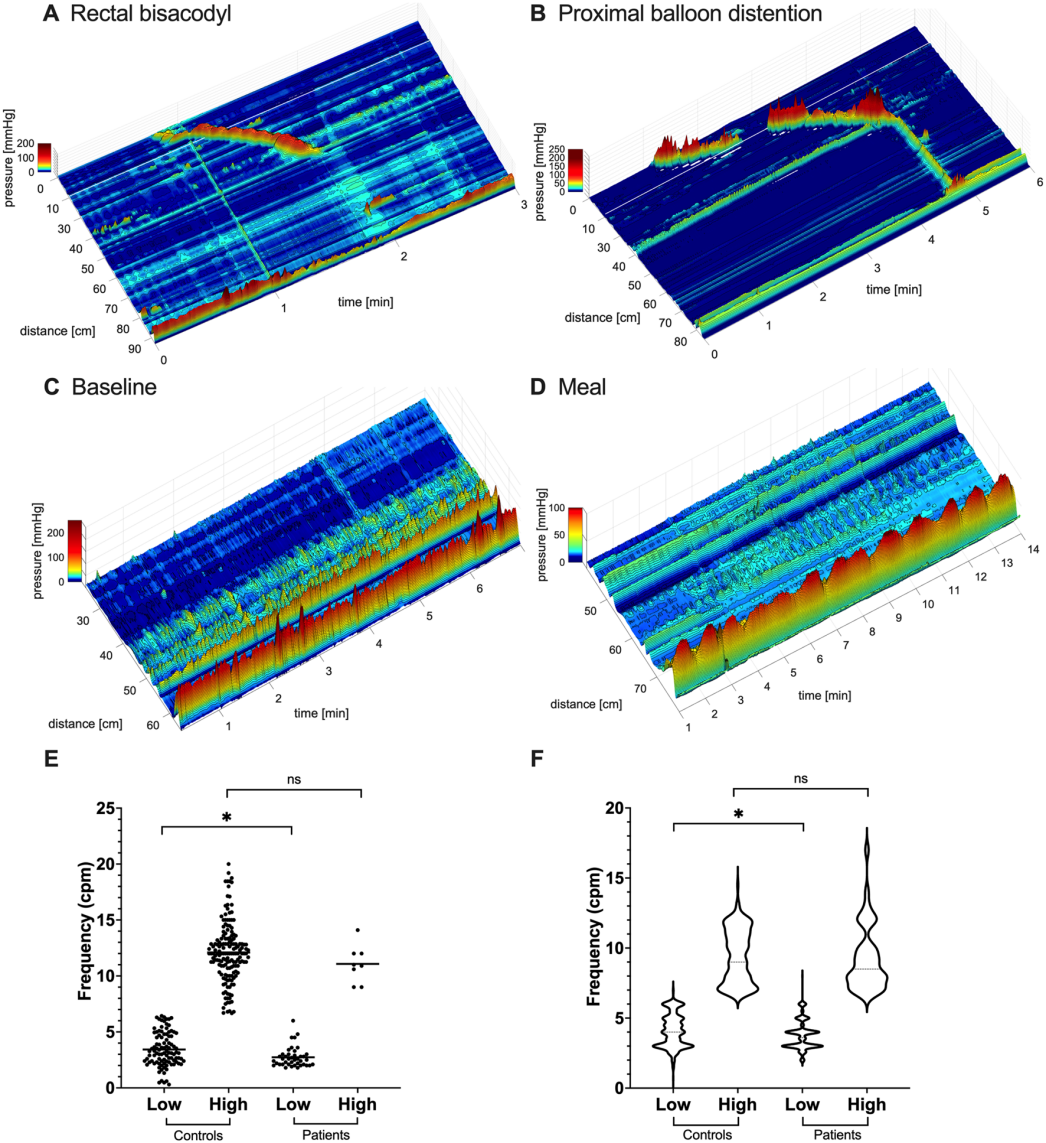
Colonic motor activity: high-amplitude propagating pressure waves (HAPWs), high and low frequency cyclic motor patterns (CMPs), and haustral activity in patients. (**A**) Rectal bisacodyl evoked an HAPW that originated in the ascending colon (at 0 cm) and propagated into the descending colon (at 42 cm) in patient #6. This shows evidence of both vagal and sacral pathway activity. Patient #6 also showed a normal coloanal reflex: HAPW associated anal sphincter (at 89 cm) relaxation without persistent sphincter of O’Beirne (at 75 cm) activity. The white line represents a gap of 10 cm where a balloon was attached to the catheter. (**B**) PBD evoked an HAPW that originated in the ascending colon (at 0 cm) in patient #4. This shows evidence of vagal pathway activity and evidence of coloanal dyssynergia: the HAPW is associated with anal sphincter contraction (at 80 cm). The white line represents a gap of 10 cm where a balloon was attached to the catheter. (**C**) During baseline, a retrograde propagating high frequency cyclic motor pattern (at 40–52 cm) was observed with sphincter of O’Beirne activity (at 55 cm) and a high anal sphincter pressure (at 63 cm) in patient #12. (**D**) In response to a meal, retrograde propagating low frequency cyclic motor patterns (at 55–62 cm) and rhythmic high amplitude anal sphincter pressure activity (at 73 cm) were observed in patient #3. (**E**) Each dot represents the average intrinsic pressure wave frequency within a single cyclic motor pattern. Mean value is shown as a black line. There were more high frequency CMPs than low frequency CMPs in controls, whereas in patients there were more low frequency CMPs compared to high frequency. Controls: low frequency (n = 99), high frequency (n = 164). Patients: low frequency (n = 42), high frequency (n = 8). (**F**) The average frequency of the low frequency haustral activity is significantly lower in the patient group (P < 0.0001). Controls: low frequency (n = 563, N = 21), high frequency (n = 160, N = 21). Patients: low frequency (n = 505, N = 14), high frequency (n = 40, N = 14). Low frequency controls vs patients: 4.1 ± 1.2 cpm vs 3.7 ± 1.0 cpm. High frequency controls vs patients: 9.4 ± 1.9 cpm vs 9.3 ± 2.3 cpm.

Rectal bisacodyl evoked a HAPW response in 8 of 13 (62%) patients, compared to 12 of 13 (92%) healthy subjects (Tables 1, 2). The amplitude of rectal bisacodyl evoked HAPWs (all HAPW types) was significantly lower in patients than in healthy subjects (P < 0.0001) (Fig. 1C, Table 1). The sacral autonomic reflex induced by rectal bisacodyl was present in 38% of patients compared to 69% of healthy subjects (Table 2), with significantly lower HAPW amplitudes in patients (71.0 mmHg vs 120.0 mmHg, P < 0.0001, Fig. 1C). One patient without the sacral autonomic reflex showed the bisacodyl evoked vagosacral reflex with HAPWs propagating into the descending colon implying left colon functioning (Patient #6 Table 3, Fig. 2A). All patients with normal HAPWs showed sacral pathway activity, whereas patients #8–14 with weak HAPWs or without HAPWs did not show activity mediated by the sacral pathway.

### Vagosacral coordination of colonic motility and anal sphincter activity

Coordination of vagal and sacral activity in HAPW generation is most clearly reflected in the vagosacral reflex, indicated by HAPWs that propagate from the proximal colon to the descending colon, combined with the coloanal reflex. The vagosacral reflex was evident in 4 of 8 (50%) patients compared to 13 of 19 (68%) healthy subjects (Tables 2, 3). Poor sacral control of coloanal coordination was unique to the patient cohort. While all healthy subjects showed a normal coloanal reflex, it occurred only in 58% of patients (P = 0.0047, Table 2). Instead of showing anal sphincter relaxation in response to a propulsive motor pattern, five patients showed no relaxation or even a paradoxical contraction of the anal sphincters (Table 3), indicative of coloanal dyssynergia, which would obstruct normal defecation (Fig. 2B). Coloanal dyssynergia was found in all three patient groups, including 3 of the 4 patients who belonged to the normal HAPW group (Table 3), indicating the dominant pathophysiology in this group. Coloanal dyssynergia was assessed in response to SPWs when no HAPW activity was present.

Taken together, the absence of efferent sacral pathway activity, shown by evaluating the vagosacral reflex and the sacral autonomic reflex, and poor colon—anal sphincter coordination all contributed to left colon dysmotility, which was present in 10 of 14 (71%) patients. Seven of the 10 patients had prior diagnostic test results of a positive balloon expulsion test, delayed left colon to pelvic region transit, or both (Patients #1, 8–13, Supplementary Table S1).

### Associations between left colon hypertonicity, sphincter of O’Beirne activity and coccyx injury

Sustained intraluminal pressure was measured as the average pressure over 6 sensors which did not record motor patterns, within 50 min of baseline activity. Patients with coccyx injury had a significantly higher sigmoid colon vs ascending/transverse colon pressure difference compared to healthy subjects (9.3 ± 0.3 (n = 4) vs. 3.8 ± 0.9 mmHg (n = 17), P = 0.021) (Supplementary Table S2). The high sigmoid colon pressure (up to 16.1 mmHg) suggests left colon hypertonicity. In addition, high sphincter of O’Beirne activity was associated with coccyx injury in patients # 1, 8, and 12 (Table 3, refer to the section “The sphincter of O’Beirne”).

### Autonomic nervous system function and its correlation with colonic dysmotility

#### General autonomic function based on HRV during supine position and reactivity to active standing

**Figure 3:**
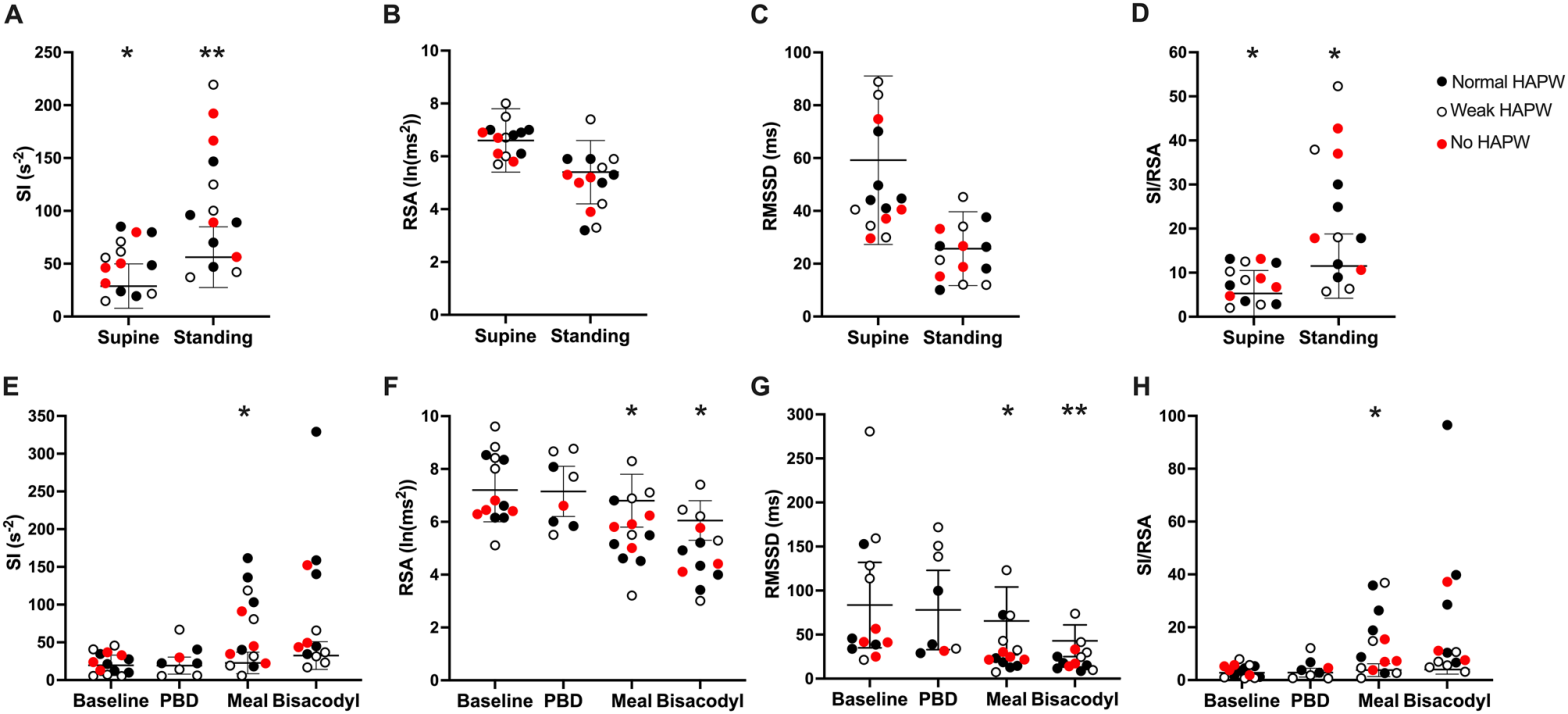
Comparison of autonomic nervous system activity in patients and healthy subjects. Patients are divided into three groups, those with normal HAPW activity (black), those with weak HAPW activity (open), those without HAPW activity (red). (**A**–**D**) Sympathetic (SI) activity, parasympathetic (RSA, RMSSD) activity, and autonomic balance (SI/RSA) during the active standing test for the assessment of general autonomic function. Active standing test controls: RSA and RMSSD N = 33, SI and SI/RSA N = 20. (**E**–**H**) Sympathetic (SI) activity, parasympathetic (RSA, RMSSD) activity, and autonomic balance (SI/RSA) during the HRCM procedure where autonomic functioning was assessed in quiescent periods during baseline and in response to stimuli. All lines represent mean ± SD of healthy subjects. HRCM controls: baseline and meal N = 10, PBD N = 8, rectal bisacodyl N = 9)^15^. Most patients show high sympathetic tone (supine) and high sympathetic reactivity in response to stimuli, from supine to standing, proximal balloon distention (PBD), 1000 kcal meal and rectal bisacodyl. Low RSA in response to the 1000 kcal meal and rectal bisacodyl was also present. SI = sympathetic index (Baevsky’s stress index). RSA = respiratory sinus arrhythmia, RMSSD = root mean square of successive differences between heartbeats. SI/RSA = Autonomic balance. High and low designations indicate values above or below 1SD of healthy subjects. Each dot represents one patient (N = 14). * P < 0.05, ** P < 0.01.

**Figure 4:**
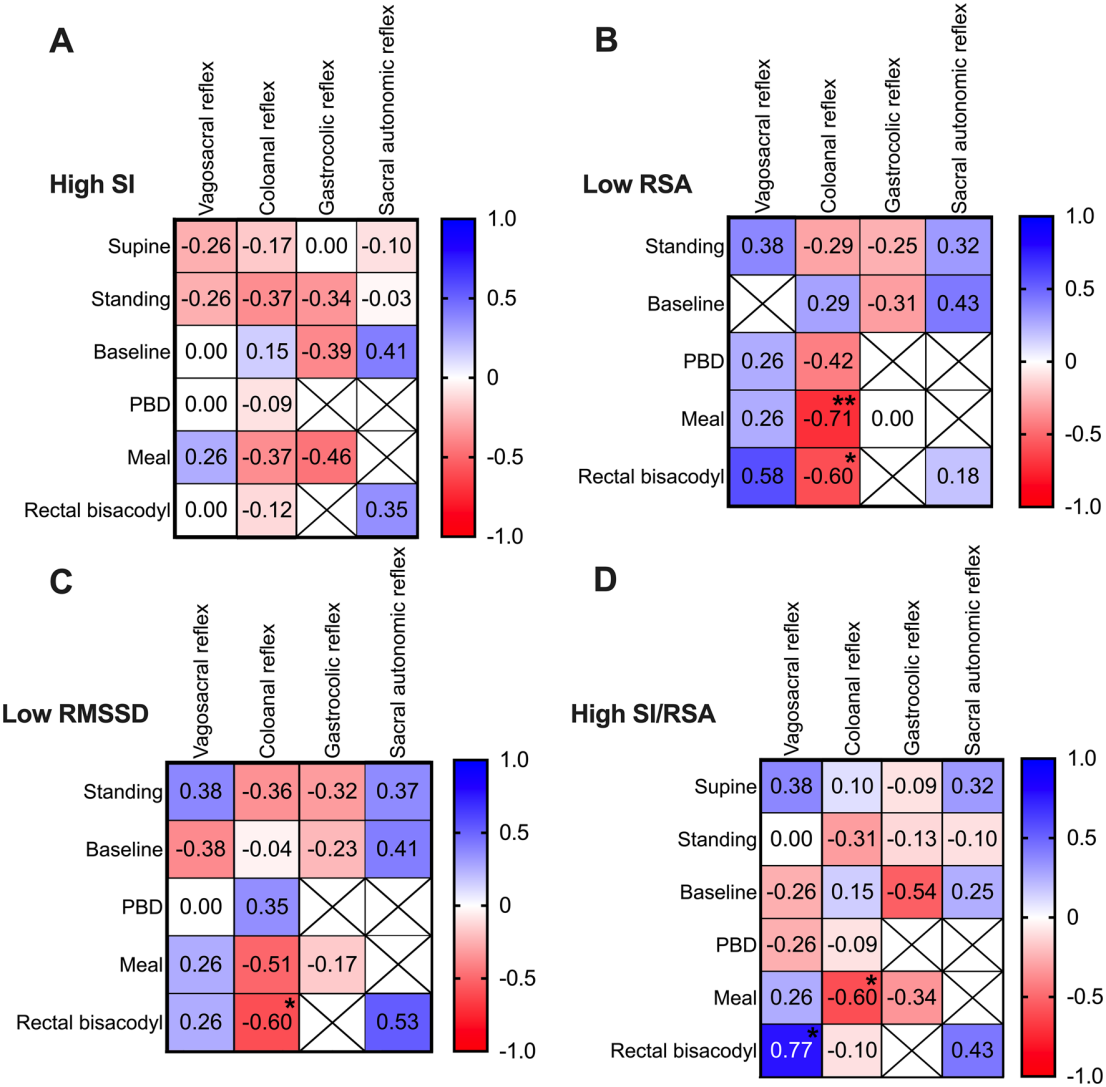
Correlation between autonomic nervous system parameters and the presence of colonic reflexes in patients with chronic constipation using a Pearson correlation matrix. We assessed (**A**) high sympathetic (SI) tone (supine and baseline) and sympathetic (SI) reactivity to stimuli, (**B**) and (**C**) low parasympathetic (RSA, RMSSD) tone (supine and baseline) and parasympathetic reactivity to stimuli, and (**D**) overall sympathetic dominance as reflected in a high ratio of SI/RSA. Low parasympathetic tone during supine did not occur in any patient, thus was excluded. Correlations that are not theoretically associated (e.g. PBD does not stimulate the gastrocolic reflex) were excluded. Presence of low RSA and low RMSSD reactivity to meal and rectal bisacodyl showed a significant negative correlation with the presence of the coloanal reflex (CAR): all patients without the coloanal reflex had low RSA reactivity. * P < 0.05, **P < 0.01.

High sympathetic (SI) reactivity to active standing was a significant feature in patients with chronic refractory constipation. High sympathetic reactivity was defined by SI values that were more than one SD above the mean of healthy controls. Sympathetic (SI) reactivity to active standing was significantly higher in patients than in healthy subjects (Fig. 3A) although it did not highly correlate with colonic reflexes (Fig. 4A).

The average parasympathetic (RSA and RMSSD) tone and reactivity to active standing were similar in patients and healthy subjects, where all patients had normal parasympathetic (RSA and RMSSD) tone (Fig. 3B,C). Low parasympathetic (RSA and RMSSD) reactivity did not significantly correlate with the absence of colonic reflexes (Fig. 4B,C). Consequently, no significant correlation was observed between autonomic balance (SI/RSA) in reactivity to active standing and colonic reflexes (Fig. 4D), although autonomic balance (SI/RSA) was significantly different from healthy subjects (Fig. 3D).

#### Autonomic reactivity and its association with autonomic motor reflexes in response to vagal and sacral stimuli

**Figure 5:**
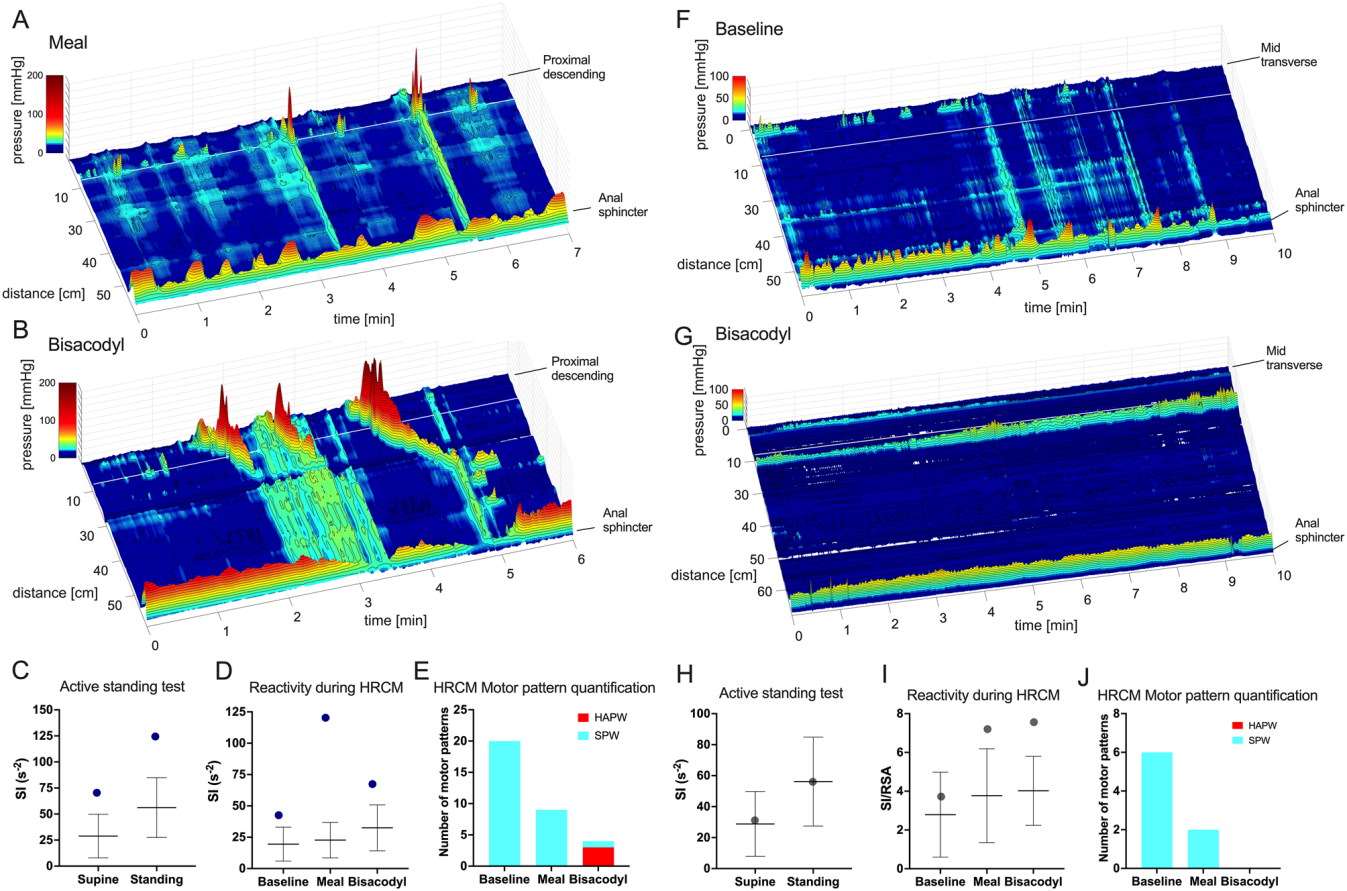
Decreased motility associated with high sympathetic tone and high sympathetic reactivity in patient #7 (**A**–**E**), and patient #12 (**F**–**J**). Sympathetic tone and reactivity are reflected by the sympathetic index SI or SI/RSA. (**A**) Patient #7 had dominant simultaneous pressure waves in response to the meal which was associated with very high sympathetic reactivity. (**B**) Rectal bisacodyl evoked a normal sacral autonomic reflex: descending colon HAPWs with a normal coloanal reflex, (**C**) this patient showed high SI tone and reactivity during the active standing test prior to HRCM, and (**D**) a decrease in SI reactivity in response to bisacodyl compared to the meal. (**E**) An increase in HAPWs occurred in response to rectal bisacodyl. (**F**) Patient #12 had a complete absence of HAPWs but SPWs were evoked during baseline when the autonomic balance (SI/RSA) was normal. (**G**) No SPWs occurred in response to rectal bisacodyl. (**H**) This patient showed normal sympathetic tone and reactivity assessed by the active standing test. (**I**) and (**J**) The decrease in SPWs in response to the meal and rectal bisacodyl was associated with high SI/RSA. The gastrocolic and the sacral autonomic reflex did not occur. The white lines represent a gap of 10 cm where a balloon was attached to the catheter.

In our cohort, 12 of the 14 patients had high sympathetic (SI) and/or low parasympathetic (RSA, RMSSD) reactivity to either vagal or sacral stimuli. Examples of the autonomic influence on motility were shown in two cases (patients #7 and 12) in Fig. 5, where decreased colonic motility was associated with high sympathetic (SI) reactivity.

Baseline sympathetic (SI) activity during the HRCM procedure in patients was overall similar to healthy subjects (Fig. 3E, Supplementary Table S3). High baseline sympathetic (SI) activity was not significantly correlated with the gastrocolic reflex (Fig. 4A). Still, patients with high baseline sympathetic (SI) activity were more likely to have an absence of the gastrocolic reflex based on a likelihood ratio of 5 (Supplementary Table S4).

The patient cohort was not statistically different from the cohort of healthy subjects in autonomic reactivity to PBD (Fig. 3E–H, Supplementary Table S3). Associations between autonomic reactivity to PBD and the presence of reflexes were weak (r < 0.5, Fig. 4), and there was no evidence of HAPW and SPW inhibition by low parasympathetic (RSA, RMSSD)/high sympathetic (SI) reactivity in three patients (patients #1, 3, 10) in response to PBD (Table 1, Supplementary Table S3).

Meal intake induced significantly higher sympathetic (SI) and/or lower parasympathetic (RSA, RMSSD) reactivity in patients (Fig. 3E–H). High sympathetic reactivity to the meal was evoked in 5 of 6 (83%) patients who did not generate the gastrocolic reflex (Table 3, Supplementary Table S3) although a statistical analysis did not find evidence of a significant correlation between high sympathetic (SI) reactivity to the meal and the gastrocolic reflex (Fig. 4A). Interestingly, low parasympathetic (RSA, RMSSD) reactivity to the meal significantly correlated with the absence of a normal coloanal reflex (Fig. 4B,C). Patients who had low parasympathetic (RSA, RMSSD) reactivity to the meal were more likely to have coloanal dyssynergia, with likelihood ratios of 3.5 and 4.8 respectively (Supplementary Table S4). In healthy volunteers, HAPW activity is associated with a significant increase in parasympathetic activity and a decrease in sympathetic activity^25^ which we interpret to reflect autonomic activity that is causing the HAPW. We did not observe significant changes in HRV parameters when HAPW activity occurred in patients (Supplementary Table S5) reflecting weaker activation of the autonomic nervous system, consistent with lower amplitudes of the HAPW. More specifically, 7 of 9 patients had low parasympathetic (RSA, RMSSD) activity and 4 of 9 patients had high sympathetic (SI) activity during HAPWs (Supplementary Table S3, Supplementary Table S5).

### Cyclic motor pattern activities at 3 cpm and 12 cpm

The human colon generates a 3 cpm cyclic motor pattern (Fig. 2D), orchestrated by interstitial cells of Cajal associated with the submuscular plexus (ICC-SMP), that serves to enhance absorption and restrict transit. This myogenic motor pattern is triggered by parasympathetic neural stimulation^26,27^. Patients showed a reduction in cyclic motor pattern activity compared to healthy controls. Healthy subjects showed 13 cyclic motor patterns per subject with a total of 250 in 19 subjects, while patients showed 4 cyclic motor patterns per subject with a total of 50 in 14 patients. Almost all (64%) colonic cyclic motor pattern activity was seen in 2 patients (patients #1 and 3) associated with normal HAPW activity. The cyclic motor patterns in the patients travelled along the colon for a significantly shorter distance than in healthy subjects for all cyclic motor pattern types (HAPW-related cyclic motor pattern: 9.1 ± 6.8 cm vs. 14.1 ± 17.4 cm, P < 0.0001; colonic cyclic motor pattern: 8.2 ± 3.4 cm vs. 17.2 ± 22.7 cm, P = 0.008).

The frequency of the 3 cpm colonic cyclic motor pattern was 3.4 ± 1.5 cpm (n = 99) in healthy controls vs 2.7 ± 0.9 cpm (n = 42) in patients; although the mean was significantly different (P = 0.003), all values in the patient group were within the range of control values (Fig. 2E).

The human colon also generates a 12 cpm cyclic motor pattern (Fig. 2C), orchestrated by interstitial cells of Cajal associated with the myenteric plexus (ICC-MP), triggered by parasympathetic neural activity, often associated with the generation of HAPWs^27^. The 12 cpm cyclic motor pattern was present in 4 patients (patients #3, 5, 9, 12), associated with HAPW activity in patients #3 and 9 (Fig. 2E, Table 1).

### Haustral activity

The human colon is compartmentalized into haustra, bounded by circular muscle, sphincter-like, boundaries. The boundaries may restrict transit, and intrahaustral cyclic motor patterns provide robust motility to enhance absorption and stool formation^28,29^. Both boundaries and the intrahaustral cyclic motor pattern show rhythmicity at ~ 3 cpm and ~ 12 cpm.

All patients showed 3 cpm haustral activity that was similar to controls. Although the average frequency was significantly lower in patients, its range was the same as in controls (Fig. 2F). The abundant presence of haustral activity indicated that no patient showed a truly inert colon. In healthy subjects, 3 cpm haustral activity occurred for a duration of 27.7 ± 21.8 min/h; in the patient cohort it occurred for 31.0 ± 22.3 min/h (P = 0.326). All periods of haustral activity are displayed in Fig. 2F, with its intrinsic pressure wave frequency showing a consistent presence of the 3 cpm frequency.

The 12 cpm haustral activity was reduced in the patient cohort and was not seen in all patients. Its duration in healthy subjects was 7.4 ± 9.1 min/h, whereas the duration was 2.2 ± 2.1 min/h (P = 0.15) in the patient cohort. The absence of 12 cpm activity in patients #13 and 14 (Table 1) together with the absence of HAPWs, strongly suggests a reduction in the ICC-MP network.

Patients did not show a significant *increase* in haustral activity; hence excessive haustral activity did not appear to be a cause of constipation.

### The sphincter of O’Beirne

Excessive activity of the sphincter of O’Beirne and lack of its relaxation during the coloanal reflex (autonomous dyssynergia) were contributors to the pathophysiology of constipation^30,31^. In 5 patients (# 1, 3, 8, 9, 12), sphincter of O’Beirne activity was higher than 1 SD from the mean of controls (present 19.3 ± 16.9% of total recording time) (Table 3); its presence was 63.2% (patient #1), 36.3% (patient #3), 49.4% (patient #8), 65.2% (patient #9), and 65.7% (patient #12) of the total time^30,32^. Its mean amplitude was 23.1 ± 9.5 mmHg across all patients, not different from 27.6 ± 7.8 mmHg (P = 0.117) in controls.

The interaction between the sphincter of O’Beirne and the anal sphincter during 32 motor patterns (HAPWs or SPWs) was assessed. A relaxation of the sphincter of O’Beirne in response to a motor pattern was seen 52% of the time, whereas a relaxation of both the anal sphincter and sphincter of O’Beirne in response to a motor pattern was only seen 37% of the time. Patients #1, 8 and 12 had no relaxation of both the sphincter of O’Beirne and anal sphincter with any motor pattern in any intervention, suggesting highly significant autonomous dyssynergia (Table 3).

### Pathophysiology of individual patients

The capabilities and inabilities of the colon and rectoanal region regarding motor pattern generation, stimulus response and associated autonomic reactivity, are reported for all patients, relevant for management. Although the statistical comparisons between the patient cohort and the controls showed important differences, each patient had a unique combination of motor patterns, abnormal reflexes and HRV parameters, hence a unique pathophysiology phenotype, hypothesized in Table 3 and Supplementary Table S1. Pathophysiological features included coloanal dyssynergia in patients #1, 2, 4, 10, and 13, absence of vagal pathway activity in patient #7, absence of sacral pathway activity in patients #8, 9, 10, and 13, and absence of both vagal and sacral pathway activity in patients #11, 12, and 14 (Table 3, Supplementary Table S1).

In patients #1, 3, 8, 9 and 12, a high overall presence of the sphincter of O’Beirne was seen in comparison to controls (Table 3). Additionally, relaxation of the sphincter in response to motor patterns was not seen all the time. A consistent sphincter of O’Beirne pressure with lack of relaxation associated with the coloanal reflex, likely contributes to pathophysiology. Only patients #8 and 9 did not have high sympathetic (SI) or low parasympathetic (RSA, RMSSD) reactivity to any HRCM stimuli; hence their pathophysiology may not include autonomic dysfunction (Fig. 3E–H, Table 3).

## Discussion

The present study is the first to investigate pan-colonic motor patterns, autonomic reflexes and quantitative autonomic functioning, in patients with chronic refractory constipation. These patients showed mild vagal pathway impairment presented as lower HAPW amplitude, but significant left colon dysmotility presented as less descending colon HAPW activity, lower left colon HAPW amplitudes and impaired coloanal coordination. Left colon hypertonicity was observed in patients with a history of coccyx injury. Patients showed high sympathetic tone, high sympathetic reactivity to active standing and to meal intake, and lower parasympathetic and higher sympathetic activity during the occurrence of HAPWs (Supplementary Table S3). Pan-colonic high-resolution manometry with HRV provided information on the capability and inability of the entire colon and associated autonomic functioning of each patient, leading to different management strategies including pharmacological and non-pharmacological treatments, such as sacral neuromodulation for left colon dysmotility.

Diagnosis of patients based on average values resulting from cohort analysis, did not do justice to any individual patient. Hence individual diagnoses were based on the occurrence and architecture of HAPWs and SPWs and on the vagal and sacral pathway function, derived from HAPW characteristics, and HRV characteristics at baseline and in response to stimuli. Analysis of the coloanal reflex, the autonomous relaxation of the anal sphincters with the occurrence of HAPWs and SPWs, was also included. Ultimately, each patient had a unique combination of motor dysfunction and ANS dysfunction; consequently, we present key features of each patient and highlight biomarkers used to arrive at the diagnosis (Table 3 and Supplementary Table S1).

HAPWs are considered the most prominent colonic motor pattern for colon transit. The inability to generate HAPWs in response to different stimuli, specifically luminal bisacodyl administration, has been historically considered a manometric biomarker for surgical intervention for severe constipation. Rectal bisacodyl can evoke HAPWs that propagate from the most proximal colon into the descending colon in healthy subjects^20^, which shows the capability to generate a vagosacral reflex. Triggering of this motor activity involves integration of spinal sensory information in the NTS, which proceeds to the vagal dorsal motor nucleus generating proximal colon motor patterns through the vagus nerve^33,34^. Our patients with refractory chronic constipation only showed mild vagal pathway impairment. There was no significant difference in the presence of rectally stimulated proximal colon HAPWs and the presence of the gastrocolic reflex between healthy subjects and patients; consistently, none of these patients had significant upper GI dysmotility such as gastroparesis or small intestinal pseudo-obstruction at the time of the HRCM. HAPWs in response to rectal bisacodyl in the absence of spontaneous or meal-induced HAPWs, shows reserve capability of the colon to produce HAPWs, although the amplitudes were lower than that of healthy controls. Dinning et al. observed poor vagosacral communication by a decrease in proximal colon propagating sequences in 7 patients with obstructive defecation after stimulating the rectum with chenodeoxycholic acid, compared to an increase shown in 10 healthy subjects; rectal chenodeoxycholic acid induced HAPWs in one patient and in one healthy subject^35^.

While the proximal colon is innervated by the vagus nerve, parasympathetic innervation to the distal colon originates from the lumbosacral spinal cord regions of S1–S4. Damage to parasympathetic nerves will cause dysregulated colonic motility^33^. Based on our autonomic function testing, in our patient cohort, the main feature associated with poor parasympathetic reactivity was coloanal dyssynergia.

We suggest the term “left colon dysmotility” for the most prominent dysmotilities observed in our patient cohort, which includes an absence of the sacral defecation reflex and coloanal dyssynergia. Dysmotility of the left colon was a common pathophysiology shown in 71% of our patients. Left colon dysmotility was prominently associated with increased sympathetic tone and/or reactivity and was observed in all HAPW groups regardless of overall HAPW activity. Spinal injury can contribute to left colon dysmotility. Damage to the cauda equina or conus medullaris, where the sacral parasympathetic nucleus resides, resulted in a decrease in descending and sigmoid colon transit time^36^. In the present study, all 5 patients with a history of coccyx injury presented with left colon dysmotility, associated with high sympathetic reactivity. In addition, hypertonicity of the left colon was observed, resulting in an increased negative pressure gradient from proximal to sigmoid colon.

Regarding left colon dysmotility, clinical biomarker symptoms include the loss of spontaneous bowel movements, difficulty in passing gas, prolonged toilet time, loss of normal caliber stool, small pellets stool or liquid stool on laxatives, and rectal fecal retention despite excessive straining. On digital rectal examination, it may be associated with poor anal sphincter contractility on squeezing, paradoxical contraction on attempted evacuation, and minimal perineal descent on pushing.

Coloanal dyssynergia was a pathophysiological finding unique to the patient group, prominent in patients with normal HAPWs. Coloanal dyssynergia often occurred with paradoxical contraction of the sphincter of O’Beirne and was highly correlated with low parasympathetic reactivity to the meal. A normal coloanal reflex involves relaxation of both the sphincter of O’Beirne and anal sphincters during the propulsive motor patterns of HAPWs and SPWs^20,37^, leading to successful defecation. Paradoxical contractions of the anal sphincters were generating pressures up to 250% from resting pressure in some patients. In patient #1, the abnormal coloanal reflex included sphincter of O’Beirne contraction and high tone instead of relaxation^30,32^. Numerous SPWs associated with coloanal dyssynergia were seen in one patient who did not generate any HAPWs. The coloanal reflex cannot be assessed through standard anorectal manometry and it is autonomous, in contrast to the intrinsically mediated rectoanal inhibitory reflex (RAIR) that is measured as a response to a conscious stimulus; thus an abnormal coloanal reflex can be referred to as autonomous dyssynergia^38,39^. Hence, the coloanal reflex is an important biomarker to assess obstructive constipation, and its evaluation should be part of any HRCM protocol. In coloanal dyssynergia, poor inhibition of anal sphincter pressure may relate to reduced parasympathetic activity on intrinsic inhibitory neurons^40,41^, reduced activity of neural nitric oxide synthase positive parasympathetic nerves^42,43^ or insufficient withdrawal of sympathetic activity^44^.

Rectal bisacodyl was an effective and rapid stimulus that revealed the capability of left colon motility (generation of HAPWs) in half the patients. Evoking an HAPW that starts in the descending colon in response to rectal bisacodyl stimulation shows evidence of an intact sacral reflex pathway^12^. We have shown rectal bisacodyl to have a high chance of evoking HAPWs that propagate through the left colon in healthy subjects^20^. In our patients without spontaneous HAPWs, and even in two patients without vagally evoked HAPWs, rectal stimulation via bisacodyl triggered activity of the sacral reflex pathway. This indicates that bisacodyl can overcome a weak reflex; it shows reserve capability to generate HAPWs^21^.

The ICC-SMP are the dominant pacemaker cells in the human colon with a frequency range of 2–4 cpm, often referred to as the 3 cpm activity^45–47^. All patients had an abundant presence of 3 cpm haustral activity, and 84% of the cyclic motor patterns that were present were also of the 3 cpm category suggesting that the ICC-SMP network is functional in the patient cohort. It is the neural activity that uses the 3 cpm pacemaker cells to generate cyclic motor patterns that are affected in chronic constipation. Hence, the dramatic reduction in the 3 cpm cyclic motor pattern appears to be related to sympathetic inhibition of enteric neural excitatory activity.

The ICC-MP are a stimulus-dependent secondary pacemaker system in the human colon^46,47^. We hypothesized that neural stimuli that initiate the HAPW evoke pacemaker activity in the ICC-MP centering on 12 cpm^26^. Based on this, the ICC-MP network was evaluated in patients by assessing the occurrence of HAPWs as well as the high frequency (10–15-cpm) cyclic motor pattern and haustral activity at this frequency. Patients #13 and 14 did not have the presence of HAPWs, nor high frequency haustral activity nor high frequency cyclic motor patterns, suggesting a reduction or impairment of the ICC-MP network.

Sympathetic innervation of the gastrointestinal tract originates from the thoracic and lumbar spinal cord, predominantly from L2 to L5. Stimulation of sympathetic nerves leads to inhibition of colonic motility via the release of norepinephrine on cholinergic nerves in the myenteric plexus and contraction of the anal sphincters via direct action on smooth muscle cells^33,48^. Colonic inhibition by sympathetic activation occurs prominently through entero-enteric reflexes whereby distention of a section of the colon or rectum inhibits activity in more proximal parts^48,49^. Our study found that high sympathetic reactivity to vagal and sacral stimuli was prevalent, observed in 12 of the 14 patients. Consistently, constipation is common in patients with postural orthostatic tachycardia syndrome (POTS)^50^.

In the present study, high sympathetic and/or low parasympathetic reactivity was found in almost all patients regardless of age. Both the number of HAPWs^51^, and the parasympathetic parameters of HRV decreases with age^52,53^, hence, pediatric patients in the present study were expected to have a more active colon with higher parasympathetic HRV features. However, only one pediatric patient did not have high sympathetic or low parasympathetic reactivity to any HRCM stimuli. Hence, the general diagnosis of sympathetic inhibition of colonic motility was evident for both pediatric and adult patients.

According to current pediatric guidelines, the only normal HAPW is one that propagates from the proximal colon into the sigmoid colon^4^. However, this should be up for discussion since we found that young, healthy adults 18–20 years had HAPWs that start in the proximal colon and end near the splenic flexure, and HAPWs that start around the splenic flexure^20^. In the present study, a full HAPW was present in 3 of the 4 pediatric patients in which the catheter reached the proximal colon, whereas none of these 4 patients showed the sacral autonomic reflex. Only 2 pediatric patients showed a HAPW that started near the splenic flexure, one of whom had coloanal dyssynergia, resulting in 7 of the 8 pediatric patients exhibiting evidence of left colon dysmotility.

Non-invasive sacral neuromodulation, such as transcutaneous electrical nerve stimulation (TENS) and lumbosacral low-level laser therapy (LLLT), are potential treatments of chronic left colon dysmotility^54,55^. Sacral nerve stimulation by implanted electrodes caused immediate changes in rectal blood flow, indicating stimulation of autonomic innervation and was shown to improve bowel function and relieve symptoms^56,57^. Electro-acupuncture has been proven successful and shown to increase parasympathetic tone based on HRV analysis^58^. Increased parasympathetic and decreased sympathetic activity was observed in rats in response to sacral nerve stimulation with implanted electrodes^59^.

In our cohort, although surgery was contemplated for all, only one patient proceeded to surgery since most patients showed the presence of some, albeit inhibited, motor patterns. Patient #11 had lost spontaneous bowel movements for more than 10 years since a coccyx injury. HRCM showed left colon dysmotility with associated high sympathetic reactivity. After a 4-month home TENS treatment, daily bowel movement recovered and remained present for the last 3 years. The overlapping bladder symptoms were also completely controlled. We hypothesize that sacral neuromodulation restored the defecation reflex and removed sympathetic excitation due to the coccygeal injury.

In summary, our HRCM and HRV study design allowed for a quantitative assessment of colonic motor functioning and ANS functioning that contributed significantly to the understanding of the heterogeneous pathophysiology of chronic refractory constipation, allowing for evidence-based optimal management of chronic constipation.

## Methods

### Patients

Fourteen patients (age 6–43; 12 females, 2 males; 6 adult, 8 pediatric (Table 1) diagnosed with chronic refractory constipation were assessed. All participants and parents/guardians of pediatric patients gave written informed consent, and all procedures were approved by the Hamilton Integrated Research Ethics Board (HiREB) and performed in accordance to relevant guidelines. Exclusion criteria included abdominal surgery, hepatic, kidney, or cardiac diseases, connective tissue disorders, central nervous system disorders, thyroid diseases, prostate diseases, or any malignancies. All subjects were diagnosed with chronic constipation based on the Rome IV criteria, responded poorly to pharmacological treatment, and were considered for surgery. Before the start of the study, subjects were briefed on details of the study and informed that there might be discomfort due to liquid expulsion during motor activity.

### High-resolution colonic manometry (HRCM)

HRCM was performed using a previously established custom-made platform^20^. In brief, one of three 84-sensor catheters was used with different balloon configurations: one balloon between sensors 10 and 11, or two balloons between sensors 10 and 11 and sensors 40 and 41, or one balloon between sensors 7 and 8. The catheter was inserted and fixed to the colonic mucosa with the assistance of colonoscopy while an endo clip was used via a fish wire tied to the end of the catheter. Subjects remained in a supine position for the duration of the study, with the exception of meal consumption when they were seated at a 45-degree angle. The subjects were asked to refrain from preventing or promoting gas or liquid expulsion and to report each instance when such an event occurred. All body movements, changes in abdominal pressure, urination, etc. were noted and used to remove artifacts during analysis.

### HRCM protocol

After the colonoscope was withdrawn, a rest period of at least 30 min was followed by a 90-min recording of baseline activity. The response to a 5-min balloon distention at the proximal colon was then investigated. The balloon was inflated until the first sensation was reported. This was followed by incremental increases in balloon volume by 60 ml until the maximum tolerated volume was achieved which was usually between 250 and 400 ml air. In each of these periods, the volume was sustained for a short period (between 2 and 3 min). The extent of the balloon inflation was determined by the subject’s level of discomfort in response to the distention. Inflation was stopped when the discomfort reached 6–7 on a 10-point scale, but such that the subject could manage the balloon distention for 5 min. After the 5-min distention, the balloon was deflated. Analysis of the response to balloon distention was performed on the 5-min period of sustained distention as well as a 15-min period after deflation. Next, a meal was given to induce the gastrocolic reflex. The meal was based on patient preference and reached 1000 kcal. Its effect was observed for 90 min. A bisacodyl (Dulcolax; Boehringer Ingelheim, Sanofi Canada, Quebec) suspension was injected in the rectum via a syringe or in the proximal colon via a medication catheter and its effect was studied for 30 min. Bisacodyl was given in 5 mg increments up to 20 mg. The bisacodyl suspension was made in saline by crushing 6 tablets, 5 mg each, with a pestle and mortar for 5 min. At the end of the study, an X-ray was taken using a portable X-ray machine to check catheter placement.

### Human colonic motor pattern analysis

Patient data were compared with data from a cohort of healthy subjects^15,16,20^, these reports describe the method that was used to calculate amplitudes of HAPWs.

#### Definitions of colonic motor patterns

*Proximal HAPW:* HAPWs which originate and terminate in the ascending colon, up to the hepatic flexure, referred to as category 1 HAPW in Table 1^20^.

*Full HAPW*: HAPWs which originate in the ascending colon but may terminate in the transverse, descending, sigmoid colon, or reach the rectum, referred to as category 2 HAPW in Table 1^20^.

*Descending HAPW*: HAPWs which originate in the transverse or descending colon with propagation ending in the descending colon, sigmoid colon, or the rectum, referred to as category 3 HAPW in Table 1^20^.

*Normal HAPW amplitude*: within ± 1 standard deviation (SD) from control average; mean amplitude > 50 mmHg.

*Weak HAPW amplitude*: below 1 SD from average but > 50 mmHg.

*LAPW*: A motor pattern that has the same configuration as an HAPW but its average amplitude is < 50 mmHg.

*Propulsive activity score*: (average HAPW amplitudes) × (number of HAPWs).

*HAPW response to physiological conditions*: presence of one or more HAPWs during baseline and/or in response to meal.

*HAPW response to stimulation*: presence of one or more HAPWs in response to a meal and/or PBD and/or rectal bisacodyl.

*Cyclic motor patterns*: 3 or 12 cycles-per-min clusters of colonic pressure waves that occur over multiple haustra.

*Haustral activity*: 3 or 12 cycles-per-min activity that occur within a haustrum or is restricted to a haustral boundary.

### Autonomic reflex analysis

A flowchart of vagal and sacral function assessment is presented in Supplementary Figs. S1 and S2. Stimulation of the vagal and sacral autonomic neuronal pathways in the colon have been reported to increase colonic motility^39,60,61^. Autonomic reflexes were identified using HAPWs and SPWs in response to stimuli. The afferent pathway was related to the site of stimulation and the efferent pathway was determined using the location of HAPW initiation and progression. Four reflexes were assessed, the gastrocolic reflex, the sacral defecation reflex, the vagosacral defecation reflex, and the coloanal reflex.

#### Definitions in colonic reflex assessments

*Sacral autonomic (defecation) reflex*: A descending HAPW evoked in response to rectal stimulation.

*Vagosacral autonomic (defecation) reflex*: A full HAPW, evoked with or without an external stimulus, which propagates into the left colon and is followed by anal sphincter relaxation.

*The gastrocolic reflex*: As baseline and meal intervention had the same duration, an overall increase in motor patterns with meal intake compared to baseline, including HAPWs and SPWs, was recognized as a normal gastrocolic reflex.

*The coloanal reflex*: An HAPW or SPW that is associated with > 30% anal sphincter relaxation^20^. In healthy subjects, anal sphincter relaxation occurred with 89% of proximal HAPWs and at least 95% of full and descending HAPWs^20^. With regards to SPWs, > 25% anal sphincter relaxation occurred in 67% of pan-colonic SPWs in healthy subjects^16^.

*Autonomic or coloanal dyssynergia*: Failure to relax the anal sphincter by > 30% and/or paradoxical contraction of the anal sphincter in response to a HAPW with or without an SPW. Failure should happen with the lack of relaxation occurring more than once per intervention or in response to more than 2 interventions, including baseline for HAPWs. There is SPW associated coloanal dyssynergia when > 33% of SPWs are not associated with 25% anal sphincter relaxation.

### Assessment of autonomic tone and reactivity

General autonomic functioning was assessed by the active standing protocol using HRV. Autonomic function was also assessed during the entire HRCM protocol. During HRCM, autonomic function was calculated from sections without major motor patterns during baseline and in response to interventions. In addition, autonomic functioning was calculated during HAPWs. The electrocardiogram (ECG) was recorded using three electrodes attached to the patient’s torso. The configuration of electrodes has been previously described^15^. All signals were recorded using MindWare impedance cardio GSC monitor. MindWare HRV 3.1 and IMP 3.1 software (MindWare Technologies Ltd., Gahanna, OH, United States) was used to generate RSA, RMSSD, RR intervals and corrections for artifacts. The HRV parameter SI was generated from the RR interval signal using MATLAB. SI was calculated as (AMo × 100%)/(2 Mo × MxDMn) where the mode (*Mo*) is the most frequent RR interval expressed in seconds. The amplitude of the mode (*AMo*) was calculated, using a 50 ms bin width, as the number of the RR intervals in the bin containing *Mo*, expressed as a percentage of the total number of intervals measured. The variability is reflected in *MxDMn* as the difference between longest (M*x*) and shortest (M*n*) RR interval values, expressed in seconds. The SI is expressed as s^−2^^23^.

#### The active standing test

General autonomic tone at supine baseline and the autonomic reflex in response to change from supine to standing were assessed within 4 weeks prior to HRCM. All patients were asked to refrain from caffeine, smoking, and heavy meal intake for at least 2 h prior to the test. During the test, patients were placed in a quiet room with normal lighting and room temperature. After a 10-min resting period, the recording started with a 6-min baseline in supine position, followed by 6-min sitting position and a 6-min standing position. The sitting data were not reported in the present study, and healthy control autonomic responses assessed using HRV were reported in^15^ and summarized in Supplementary Table S3.

#### Heart rate variability analysis during HRCM

HRV parameters were analyzed segments of at least 10 min during each intervention where no HAPWs or SPWs were present (quiet periods). For RSA, very rare segments with a respiration frequency outside of the 0.12–0.40 Hz range were removed from analysis. HRV in response to stimuli during interventions was analyzed from quiet periods within each intervention. Autonomic responses of healthy controls during HRCM were reported in^15^ and summarized in Supplementary Table S3.

#### Definitions in heart rate variability

*Autonomic tone:* Values of HRV parameters during the baseline supine period of the active standing test.

*Autonomic reactivity:* Response of HRV parameters to standing during active standing test, and to colonic stimuli during HRCM where no propulsive motor activity occurred.

RSA: Respiratory sinus arrhythmia, taken as a measure of parasympathetic tone or reactivity (response to stimuli).

SI: the Baevsky index, or Sympathetic Index, taken as a measure of sympathetic tone or reactivity (response to stimuli)^15,23^.

RMSSD: Root mean square of successive differences between each RR peak, as a measure of parasympathetic tone or reactivity (response to stimuli).

High or low HRV parameters in patients were defined as values greater or less than 1SD from the mean of healthy subjects^15,25^ and reported in Supplementary Tables S1 and S3. The underlying hypothesis was that inhibition of HAPW activity is due to high sympathetic activity and/or low parasympathetic activity as outlined in the introduction. We used the terms high and low and not the term “abnormal” since to identify parameters as abnormal will require more evidence including a larger database of normal values. Nevertheless, with consistent values outside of 1 SD from normal average values (parasympathetic low and/or sympathetic high) we use the term “autonomic dysfunction” to formulate a hypothesis for pathophysiology as indicated Table 3 and Supplementary Table S1. When patients have a dominant low parasympathetic activity and high sympathetic activity resulting in a high SI/RSA we hypothesize that high sympathetic tone and reactivity constitutes autonomic dysfunction as a cause of constipation. In a few patients, during different interventions, as outlined in Table 1, we observed a high parasympathetic activity. In such cases, it is important to observe whether changes in parasympathetic activity or changes in sympathetic activity are dominant, and this shows the value of SI/RSA. If parasympathetic activity is high, but SI/RSA is high, we hypothesize autonomic dysfunction based on a dominant high sympathetic activity. If parasympathetic activity is high and SI/RSA is normal or low, we do not consider this autonomic dysfunction since these conditions unlikely hinder the generation of HAPWs.

### Statistical analysis

Data sets were assessed for normal distribution using the Shapiro–Wilk normality test. The Welch’s T-test was used for normal distributions and Mann Whitney test was used for non-normal distributions in the comparison of motor pattern characteristics. Statistical tests were run using GraphPad Prism version 9. To analyze difference between proportions, Chi-squared test was used for sample sizes greater than 20 and Fischer exact tests were used for smaller sample sizes. HRV parameter changes from before to during and during to after HAPWs were assessed using either ANOVA followed by Bonferroni multiple comparison test for parametric distribution, or Friedman test followed by Dunn’s multiple comparison test for non-parametric distributions. Two tailed Pearson-correlation was used to correlate HRV parameters and the presence of reflexes. P values < 0.05 were considered to indicate statistically significant differences.

The likelihood ratio has been used in screening tests to determine whether patients with a particular disease are more likely to have a particular outcome than patients without that particular disease, where ratios greater than 1 indicate association with disease and ratios greater than 10 indicate strong evidence^62^. The likelihood ratio was calculated as the probability of finding high SI, low RSA, low RMSSD, or high SI/RSA in patients who did not evoke a reflex divided by the probability of finding the same in patients who evoked a reflex.

HAPW characteristics of healthy subjects were obtained from our previously published data of 19 healthy subjects^20^. The published HAPW amplitude ± standard error values were used to calculate standard deviation and control range of HAPW amplitude for each intervention^20^. From the same control group, HAPW propulsive activity was calculated for each healthy subject using their individual mean HAPW amplitude and number of HAPWs. Control ranges of SI, RSA, and HR of active standing test was calculated from the mean ± standard deviation of 33 healthy subjects (previously unpublished); control range of HRV parameters during individual HRCM intervention quiet periods was calculated from 10 healthy subjects (previously unpublished). HRV changes during HAPWs in healthy subjects were obtained from 11 healthy subjects as reported previously^15,25^.

## Supplementary Information

Supplementary Information.

## Data Availability

Data sets analyzed in the study are available from the corresponding author upon request.

## Abbreviations

HRCM: High-resolution colonic manometry
HAPW: High-amplitude propagating pressure wave
SPW: Simultaneous pressure wave
ANS: Autonomic nervous system
HRV: Heart rate variability
SI: Baevsky’s stress index (sympathetic index)
RSA: Respiratory sinus arrhythmia
RMSSD: Root mean square of successive differences between heartbeats
PBD: Proximal colon balloon distention
CAR: Coloanal reflex
VSR: Vagosacral reflex
GCR: Gastrocolic reflex
SAR: Sacral autonomic reflex
ICC: Interstitial cells of Cajal
