# Supplementary material for: Diagnosis of colonic dysmotility associated with autonomic dysfunction in patients with chronic refractory constipation
Source: Sci Rep. 2022 Jul 14;12:12051. doi: 10.1038/s41598-022-15945-6 (PMC9283508; doi:10.1038/s41598-022-15945-6)
Supplement: Supplementary file 1 — Supplementary Information. [file 41598_2022_15945_MOESM1_ESM.pdf]

Diagnosis of colonic dysmotility associated with autonomic dysfunction in patients with chronic refractory constipation

Lijun Liu, Natalija Milkova, Sharjana Nirmalathasan, M. Khawar Ali, Kartik Sharma, Jan D. Huizinga and Ji-Hong Chen

Supplementary Table S1.

Individual diagnoses based on autonomic reflexes, HRV responses, and past examination results of each patient

<table>
<tr><th>F/M age
HAPW</th><th>Past examination results and history</th><th>Active standing test HRV</th><th></th><th>Baseline</th><th>PBD</th><th>Meal</th><th>Bisacodyl</th><th>CMP and haustral frequency analysis</th><th>Pathophysiology hypothesis + summary</th></tr>
<tr><td rowspan="2">P-1
F43
Normal
HAPWs</td><td rowspan="2">- Chronic constipation with 2-3 bowel movements a month
- Coccyx injury
- Weak resting and squeeze pressure on anorectal manometry
- Positive balloon expulsion test
- Delayed left colon and pelvic region transit</td><td rowspan="2">Supine: Normal
Standing:
↓ RSA
↓RMSSD
↑ SI
↑ SI/RSA</td><td>HRV</td><td>Normal</td><td>↓ RSA</td><td>↑ SI
↓ RSA
↓ RMSSD
↑ SI/RSA</td><td>↓ RSA
↓ RMSSD
↑ SI/RSA</td><td rowspan="2">- 3cpm and 12cpm haustral activity present
- CMPs present</td><td rowspan="2">Hypothesis: General high sympathetic reactivity. Absent gastrocolic reflex and coloanal dyssynergia associated with high colon sympathetic reactivity.

Summary with past examination results: Dyssynergia associated with coccyx injury, weak anal sphincter pressures with positive balloon expulsion test, and delayed left colon and pelvic region transit.</td></tr>
<tr><td>Autonomic Reflexes</td><td colspan="4">- Presence of vagal and sacral pathway activity with sacral stimulated VSR and SAR
- Spontaneous HAPWs
- CAR dyssynergia and absence of GCR
- Autonomous dyssynergia with high SoB activity</td></tr>
<tr><td rowspan="2">P-2
M17
Normal
HAPWs</td><td rowspan="2">- Chronic constipation since age 9
- Paradoxical push with partial relaxation on digital examination
- Normal transit time on transit study with medication</td><td rowspan="2">Supine: Normal
Standing: Normal</td><td>HRV</td><td>↓ RMSSD</td><td>--</td><td>↑ SI
↓ RSA
↓ RMSSD
↑ SI/RSA</td><td>↑ SI
↓ RSA
↓ RMSSD
↑ SI/RSA</td><td rowspan="2">- 3cpm and 12cpm haustral activity present
- CMP not present</td><td rowspan="2">Hypothesis: Normal reflex pathways. Coloanal dyssynergia associated with high colon sympathetic reactivity.

Summary with past examination results: Dyssynergia associated with paradoxical push and partial relaxation in digital examination.</td></tr>
<tr><td>Autonomic Reflexes</td><td colspan="4">- Presence of vagal and sacral pathway activity with SAR and GCR
- Spontaneous HAPWs
- CAR dyssynergia</td></tr>
<tr><td rowspan="2">P-3
F40
Normal
HAPWs</td><td rowspan="2">- Chronic constipation with one bowel movement every two weeks</td><td rowspan="2">Supine: Normal
Standing: Normal</td><td>HRV</td><td>↑ SI
↑SI/RSA</td><td>↑ SI
↓ RSA
↓ RMSSD
↑ SI/RSA</td><td>Normal</td><td>↓ RSA
↓ RMSSD
↑ SI/RSA</td><td rowspan="2">- 3cpm and 12cpm haustral activity present
- CMPs present</td><td rowspan="2">Hypothesis: Normal reflex pathways. Low parasympathetic activity and consequently dominant sympathetic balance may reduce colonic motility, and autonomous dyssynergia may cause defecation difficulties.</td></tr>
<tr><td>Autonomic Reflexes</td><td colspan="4">- Presence of vagal and sacral pathway activity with vagal stimulated VSR, GCR, SAR, and CAR
- No spontaneous HAPWs or SPWs
- Autonomous dyssynergia with high SoB activity</td></tr>
<tr><td>P-4
F6
Normal
HAPWs</td><td>- Chronic constipation with one bowel movement every 3-5 weeks</td><td>Supine:
↑ SI
↑ SI/RSA</td><td>HRV</td><td>↑ RSA
↑ RMSSD</td><td>Normal</td><td>↑ SI
↓ RSA
↓ RMSSD
↑ SI/RSA</td><td>↑ SI ↓ RSA
↓ RMSSD
↑ SI/RSA</td><td>- 3cpm and 12cpm haustral activity present</td><td>Hypothesis: General high sympathetic tone and reactivity. Normal reflexes except coloanal dyssynergia associated with high colon sympathetic reactivity.</td></tr>
</table>

		Standing:<br>↑ SI<br>↑ SI/RSA	Autonomic Reflexes	- Presence of vagal and sacral pathway activity with vagal and sacral stimulated VSR and GCR<br>- CAR dyssynergia and no spontaneous HAPWs and SPWs				- CMP not present	
**P-5**<br>**F8**<br>Normal HAPWs	- Chronic constipation with daily soiling and inability to regularly pass stool<br>- Weak resting and squeeze pressure on anorectal manometry<br>- Positive balloon expulsion test<br>- No symptom improvement after biofeedback training<br>- Normal colonic transit time	Supine:<br>↑ SI<br>↑ SI/RSA<br>Standing:<br>↑ SI	HRV	--	--	↑ SI<br>↓ RSA<br>↓ RMSSD<br>↑ SI/RSA	↑ SI<br>↓ RSA<br>↓ RMSSD<br>↑ SI/RSA	- 3cpm and 12cpm haustral activity present<br>- CMPs present	**Hypothesis:** General high sympathetic tone and reactivity. Normal reflexes. Low colon parasympathetic reactivity and high colon sympathetic reactivity may reduce colonic motility.<br><br>**Summary with past examination results:** Vagal and sacral pathway present associated with normal transit time but weak anal sphincter pressure with positive balloon expulsion test.
			Autonomic Reflexes	- Presence of vagal and sacral pathway activity with SAR, GCR, and CAR<br>- No spontaneous HAPWs and SPWs					
**P-6**<br>**M11**<br>Weak HAPWs	- Chronic constipation since age 3<br>- Weak squeeze and ineffective bearing down on anorectal manometry<br>- Looped left colon and megarectum on x-ray<br>- Delayed left colon to rectum transit	Supine:<br>↑ RSA<br>Standing:<br>↑ RSA<br>↑RMSSD	HRV	↓ SI<br>↑ RSA	↓ SI<br>↑ RSA<br>↑ RMSSD<br>↓ SI/RSA	Normal	↓ RSA<br>↑ SI/RSA	- 3cpm and 12cpm haustral activity present<br>- CMP not present	**Hypothesis:** Weak HAPW activity associated with high colon sympathetic reactivity.<br><br>**Summary with past examination results:** Vagal and sacral pathway present with weak HAPWs and no response to PBD, associated with delayed left colon to rectum transit.
			Autonomic Reflexes	- Presence of vagal and sacral pathway activity with sacral stimulated VSR, GCR (SPWs), and CAR.<br>- No HAPW or SPW response to PBD, and no spontaneous HAPWs and SPWs					
**P-7**<br>**F24**<br>Weak HAPWs<br><br>Figure 5A-E	- Lifelong constipation with up to 2 weeks without spontaneous bowel movement<br>- Normal resting and squeeze pressure on anorectal manometry<br>- Positive balloon expulsion test	Supine:<br>↑ SI<br>↑ SI/RSA<br>Standing:<br>↓ RSA<br>↑ SI<br>↑ SI/RSA	HRV	↑ SI<br>↓ RSA<br>↓ RMSSD<br>↑ SI/RSA	--	↑ SI<br>↓ RSA<br>↓ RMSSD<br>↑ SI/RSA	↑ SI<br>↓ RMSSD<br>↑ SI/RSA	- only 3cpm haustral activity present<br>- no 12cpm haustral activity<br>- CMP not present	**Hypothesis:** General high sympathetic tone and reactivity. Weak HAPW activity and no vagal pathway activity associated with high colon sympathetic reactivity.<br><br>**Summary with past examination results:** Presence of sacral pathway activity associated with normal anal sphincter pressures, but positive balloon expulsion test.
			Autonomic Reflexes	- Presence of sacral pathway activity with SAR, and CAR,<br>- Spontaneous SPWs<br>- No vagal pathway activity with absence of GCR<br>- No HAPW or SPW response to PBD and no spontaneous HAPWs					
**P-8**<br>**F15**<br>Weak HAPWs	- Lifelong constipation with up to 2 weeks without a bowel movement<br>- Coccyx injury<br>- Positive balloon expulsion test	Supine:<br>↑ SI<br>Standing:<br>↑ SI<br>↑ SI/RSA	HRV	↑ RSA<br>↑ RMSSD	↑ RMSSD	Normal	↑ RSA<br>↑ RMSSD	- 3cpm and 12cpm haustral activity present<br>- CMP not present	**Hypothesis:** General high sympathetic tone and reactivity. Weak HAPW activity; High parasympathetic reactivity may obscure sympathetic dysfunction.<br><br>**Summary with past examination results:** Distal colon dysmotility associated with coccyx injury and positive balloon expulsion test.
			Autonomic Reflexes	- Presence of vagal pathway activity with GCR and CAR<br>- No sacral pathway activity with absence of vagal and sacral stimulated VSR, and SAR<br>- No HAPW or SPW response to PBD, and no spontaneous HAPWs and SPWs<br>- Autonomous dyssynergia with high SoB activity					
**P-9**<br>**F35**<br>Weak HAPWs	- Lifelong constipation with outlet dysfunction<br>- Weak squeeze pressure on anorectal manometry<br>- Positive balloon expulsion test<br>- Puborectalis dyskinesis at initiation of defecation	Supine:<br>Normal<br>Standing:<br>Normal	HRV	↓ SI<br>↑ RSA<br>↓ RMSSD	↓ SI<br>↑ RSA<br>↓ RMSSD<br>↓ SI/RSA	↓ SI<br>↑ RSA<br>↓ RMSSD<br>↓ SI/RSA	Normal	- 3cpm and 12cpm haustral activity present<br>- CMPs present	**Hypothesis:** No obvious autonomic nervous system dysfunction. High parasympathetic reactivity may obscure sympathetic dysfunction.<br><br>**Summary with past examination results:** Distal colon dysmotility associated with weak anal sphincters with positive balloon expulsion test, and puborectalis dyskinesis.
			Autonomic Reflexes	- Presence of vagal pathways activity with GCR and CAR<br>- No sacral pathway activity with absence of VSR and SAR<br>- No HAPW response to PBD , rectal bisacodyl, and spontaneous HAPWs and SPWs<br>- Autonomous dyssynergia with high SoB activity					

**P-10**<br>**F13**<br>Weak<br>HAPWs	- Chronic constipation with daily abdominal pain<br>- Delayed transit in left colon and pelvic region<br>- Sigmoid redundancy on x-ray	Supine:<br>↑ SI<br>Standing:<br>↑ SI	HRV	↑ SI<br>↑ SI/RSA	↑ SI<br>↓ RSA<br>↑SI/RSA	↑ SI<br>↓ RSA<br>↑SI/RSA	↓ RSA<br>↓ RMSSD	**-** 3cpm and 12cpm haustral activity present<br>-CMPs not present	**Hypothesis:** General high sympathetic reactivity. Weak HAPW activity; no autonomic reflexes associated with high colon sympathetic reactivity.<br><br>**Summary with past examination results:** Distal colon dysmotility associated with delayed left colon transit and sigmoid redundancy.
			Autonomic Reflexes	- Presence of vagal pathway activity with ascending to transverse colon HAPW during PBD<br>- Spontaneous SPWs with coloanal dyssynergia<br>- No sacral pathway activity with absence of VSR and SAR<br>- Absence of GCR, and no spontaneous HAPWs					
**P-11**<br>**F24**<br>No<br>HAPWs	- Long standing constipation with no bowel movement up to 4 weeks<br>- Coccyx injury<br>- Weak squeeze and partial anal sphincter relaxation on anorectal manometry<br>- Positive balloon expulsion test<br>- Delayed transit in left colon and pelvic region<br>- Tight narrowing in distal sigmoid on barium enema	Supine:<br>Normal<br>Standing:<br>↑ SI<br>↑ SI/RSA	HRV	↓ RMSSD<br>↑SI/RSA	↓ RMSSD<br>↑SI/RSA	↓ RMSSD	↑ SI<br>↓ RSA<br>↓ RMSSD<br>↑ SI/RSA	**-** 3cpm and 12cpm haustral activity present<br>- CMP not present	**Hypothesis:** General high sympathetic reactivity. No HAPWs and no vagal and sacral reflexes associated with high colon sympathetic reactivity.<br><br>**Summary with past examination results:** Proximal and distal colon dysmotility associated with coccyx injury, weak anal sphincters with positive balloon expulsion test, delayed left colon transit, and narrowing of distal sigmoid colon.
			Autonomic Reflexes	- No vagal or sacral pathway activity with absence of HAPW or SPW response to all stimuli and no spontaneous HAPW or SPW					
**P-12**<br>**F15**<br>No<br>HAPWs<br><br>Figure 5F-J	- Chronic constipation with Malone antegrade colonic enema procedure already performed<br>- Coccyx injury<br>- Weak resting and squeeze pressure on anorectal manometry<br>- Positive balloon expulsion test<br>- Persistent paradoxical puborectalis contraction on defecogram<br>- Normal colon transit time<br>- Small caliber sigmoid colon on single contrast study	Supine:<br>Normal<br>Standing:<br>Normal	HRV	Normal	--	↑ SI<br>↑SI/RSA	↑SI/RSA	**-** 3cpm and 12cpm haustral activity present<br>- CMPs present	**Hypothesis:** No HAPW activity and no autonomic reflexes with autonomous dyssynergia associated with high colon sympathetic reactivity.<br><br>**Summary with past examination results:** Distal colon dysmotility associated with coccyx injury, small caliber sigmoid colon, weak anal sphincters with positive balloon expulsion test, and paradoxical puborectalis muscle.
			Autonomic Reflexes	- Presence of spontaneous SPWs<br>- No vagal or sacral pathway activity with absence of HAPW response to all stimuli, and absence of GCR<br>- No spontaneous HAPWs<br>- Autonomous dyssynergia with high SoB activity					
**P-13**<br>**F36**<br>No<br>HAPWs	- Chronic constipation of 6-year duration with 1-3 bowel movements every 2 weeks with medication<br>- coccyx injury<br>- delayed transit in left colon to pelvic region	Supine:<br>↑ SI<br>Standing:<br>↓ RSA<br>↑ SI<br>↑ SI/RSA	HRV	Normal	--	↓ RSA<br>↓ RMSSD<br>↑SI/RSA	↓ RSA<br>↓ RMSSD<br>↑ SI/RSA	- only 3cpm haustral activity present<br>- no 12cpm haustral activity<br>- CMP not present	**Hypothesis:** General high sympathetic tone and reactivity. No HAPW activity, no sacral defecation reflex, and coloanal dyssynergia associated with high colon sympathetic and low colon parasympathetic reactivity.<br><br>**Summary with past examination results:** Proximal and distal colon dysmotility with poor anal sphincter coordination associated with coccyx injury and delayed left colon transit.
			Autonomic Reflexes	- Presence of spontaneous SPWs and vagal pathway activity with GCR<br>- No HAPW response to all stimuli or spontaneous HAPWs<br>- No sacral pathway activity with absence of SAR, and presence of CAR dyssynergia					
**P-14**<br>**F9**<br>No<br>HAPWs	- Chronic constipation since age 4 with up to 18 days of no bowel movement<br>- Ileostomy has already been performed with poor outcome	Supine:<br>↑ SI<br>↑ SI/RSA<br>Standing:<br>↑ SI	HRV	↑ SI<br>↑ SI/RSA	--	↑ SI<br>↓ RMSSD<br>↑SI/RSA	--	- only 3cpm haustral activity present<br>- no 12cpm haustral activity<br>- CMP not present	**Hypothesis:** General high sympathetic tone and reactivity. No HAPW activity and no gastrocolic reflex associated with high colon sympathetic reactivity
			Autonomic Reflexes	- No vagal or sacral pathway activity with absence of HAPW or SPW response to all stimuli<br>- Absence of spontaneous HAPW and SPW					

The HRCM data are formulated based on the presence and strength of the HAPWs and the presence of the coloanal reflex. The HRCM data are also interpreted as reflexes as outlined in the text.
GCR = gastrocolic reflex = response to a meal by an increase in HAPWs and/or SPWs as compared to baseline
VSR = vagosacral reflex = proximal colon originating HAPW or LAPW that travels into the descending colon and is evoked with or without an external stimulus
CAR = coloanal reflex = anal sphincter relaxes by more than 30% when associated with HAPW and LAPWs, or more than 25% when associated with SPWs. Failed relaxation associated with more than one HAPW in one intervention, one or more HAPWs in all interventions, or more than 33% of SPWs when fewer than two HAPWs are present indicates coloanal dyssynergia.
Autonomous dyssynergia - Involuntary contraction (relaxation failure) of the anal sphincters with a possible contribution of contractions of the sphincter of O'Beirne in response to a motor pattern
The up and down arrows are based on the data from Supplementary Table 3. It indicates a weak or strong response based on the value being outside 1 standard deviation from the mean.
Patients with normal HAPWs have a HAPW propulsive activity score within one standard deviation from the healthy subjects' mean value. Weak HAPWs indicate a propulsive activity score that is less than one standard deviation below the mean.

Abbreviations:
RSA = respiratory sinus arrhythmia, an indicator of parasympathetic activity
SI = sympathetic index, an indicator of sympathetic activity
SI/RSA = autonomic balance
SoB = sphincter of O'Beirne
CMP = cyclic motor patterns
cpm = cycles per minute

Supplementary Table S2. Proximal and distal colonic tonic pressure during baseline of HRCM in healthy subjects (N=17), all patients with chronic constipation (N=11), and patients with both constipation and coccyx injury (N=4)

	Healthy subjects	All patients	Patients with coccyx injury
Ascending to transverse colon tonicity (mmHg)	5.5 ± 0.6	3.8 ± 1.1	3.0 ± 1.7
Sigmoid colon tonicity (mmHg)	9.2 ± 1.0	9.1 ± 1.6	12.3 ± 1.7
Sigmoid and ascending/transverse colon pressure difference (mmHg)	3.8 ± 0.9 *	5.3 ± 1.2	9.3 ± 0.3 *

Measurements are reported as mean ± SEM measured over 6 manometric sensors (~6 cm) and through 50 min of baseline HRCM. Statistical significance is determined using Welch's T-test for parametric distributions and Mann-Whitney test for non-parametric distributions. * distal-proximal colonic pressure difference is significantly different between healthy subjects and patients with a history of tailbone injury (P=0.0208).

Supplementary Table S3. HRV characteristics of all patients in comparison with healthy subjects

Study interventions	Healthy subjects Range of control values	Patient number Sex(F/M), Age													
		P-1 F43	P-2 M17	P-3 F40	P-4 F6	P-5 F8	P-6 M11	P-7 F24	P-8 F15	P-9 F35	P-10 F13	P-11 F24	P-12 F15	P-13 F36	P-14 F9
Supine and active standing HRV															
**Supine**	RSA [5.4-7.8]	6.8	7.0	6.9	7.0	6.1	8.0	5.7	6.0	7.5	6.7	6.9	6.7	5.8	6.1
	SI [7.9-49.8]	23.9	19.4	48.7	85.2	79.9	21.7	71	61.5	14.8	55.8	46.2	31.5	50.3	79.9
	RMSSD [27.3-91.9]	70.1	44.7	44.1	49.7	41.0	88.9	30.0	34.4	84.0	40.5	40.5	74.8	37.1	29.6
	HR [55-76]	60.6	73	64.9	101	80	67.6	75	73	52.3	80.86	69.8	70	73	80
	SI/RSA [0.1-10.5]	3.5	2.8	7.1	12.2	13.1	2.7	12.5	10.25	2.0	8.3	6.7	4.7	8.7	13.1
**Standing**	RSA [4.2-6.6]	3.2	5.3	5.9	5.9	5.0	7.4	3.3	4.2	5.9	5.57	5.2	5.3	3.9	5.0
	SI [27.5-84.9]	96.1	47.0	70.1	146.8	89.1	42.1	125	219.5	37.3	100.3	192.2	56.4	166.5	89.1
	RMSSD [11.7-39.7]	10.1	26.4	26.7	37.6	18.2	45.3	12.1	12.0	34.1	21.4	15.2	26.7	33.2	18.8
	HR [70-93]	73.2	80	79.9	107.3	93	82.1	87	105	66.7	104	88.3	84	88	93
	SI/RSA [4.2-18.8]	30.0	8.9	11.9	24.9	17.8	5.7	37.9	52.3	6.3	18.0	37.0	10.6	42.7	17.8
HRV during HRCM: baseline and in response to stimuli															

**Baseline**	RSA [6.0-8.4]	6.15	6.15	6.6	8.52	N/A^{++}	8.41	5.1	9.6	8.83	8.0	6.28	6.4	6.8	6.44
	SI [6.0-33]	21.21	27.6	34.6	9.94	N/A	5.28	40.7	7.2	5.39	45.6	32.9	23.78	12.9	36.78
	RMSSD [35-132]	45.42	33.53	38.3	152.6	N/A	128.1	21.08	280.4	159.0	113.4	24.7	40.83	56.27	41.34
	HR [50.2-64.1]	57.8	78	64.9	60.59	N/A	53.84	69.0	50.5	42.79	91.1	71.34	76.40	46.4	83.56
	SI/RSA [0.6-4.98]	3.45	4.48	5.25	1.17	N/A	0.63	7.94	0.74	0.61	5.69	5.23	3.71	1.86	5.71
**PBD**	RSA [6.2-8.1]	5.83	N/A	6.0	8.07	N/A	8.66	N/A	7.7	8.76	5.5	6.6	N/A	N/A	N/A
	SI [7.9-30.5]	22.1	N/A	40.5	22.41	N/A	6.05	N/A	14.1	5.47	66.8	30.0	N/A	N/A	N/A
	RMSSD [33-123]	38.75	N/A	28.7	99.56	N/A	150.5	N/A	138.2	171.6	33.79	31.21	N/A	N/A	N/A
	HR [50.5-68]	60.7	N/A	69.4	74.6	N/A	58.6	N/A	51.8	43.3	102.6	74.2	N/A	N/A	N/A
	SI/RSA [1.13-4.49]	3.79	N/A	6.74	2.78	N/A	0.70	N/A	1.83	0.625	12.07	4.58	N/A	N/A	N/A
**Meal**	RSA [5.8-7.8]	4.61	5.15	6.8	5.48	4.51	6.88	3.2	7.1	8.29	5.5	5.8	6.23	5.0	5.90
	SI [8.5-36.9]	40.1	135.9	18.0	103.2	161.5	31.63	118.6	19.1	5.87	80.7	22.2	44.96	34.6	91.03
	RMSSD [27-104]	18.37	14.23	72.0	22.93	12.51	42.61	7.075	71.37	122.8	32.16	24.36	29.99	21.3	21.14
	HR [58-73]	69.9	108.2	81.5	107	107	84.32	95.2	76.7	53.2	105.3	82.7	87.86	64.4	103.5
	SI/RSA [1.35-6.19]	8.71	26.36	2.64	18.82	35.82	4.59	36.8	2.67	0.71	14.77	3.80	7.2	6.89	15.41
**Bisacodyl**	RSA [5.3-6.8]	4.33	4.91	5.2	3.99	3.41	5.28	6.2	7.4	6.45	3.0	4.1	5.76	4.4	N/A
	SI [14.2-50.8]	44.8	140.3	34.4	158.7	329	36.73	65.6	23.2	31.32	16.7	152.3	43.59	49.3	N/A

	RMSSD [25-61]	17.57	11.36	24.7	14.87	8.28	29.49	24.17	73.42	41.23	9.46	13.68	32.88	16.88	N/A
	HR [60-85]	67.45	115.9	77.7	107.1	105	97.24	88.5	72.1	70.4	134.0	97.8	85.55	68.3	N/A
	SI/RSA [2.24-5.81]	10.35	28.57	6.65	39.80	96.52	6.96	10.6	3.15	4.86	5.56	37.17	7.56	11.1	N/A
HRV associated with HAPWs															
**During HAPWs**	RSA [6.1-7.7]	5.03	5.96	5.80	4.35	N/A	4.00	5.94	8.15	7.60	5.22	N/A	N/A	N/A	N/A
	SI [0-110.6]	56.91	115.39	66.80	242.63	N/A	106.8	123.48	15.90	14.33	179.59	N/A	N/A	N/A	N/A
	RMSSD [33.4-99.2]	21.53	23.14	21.41	13.55	N/A	10.07	23.97	112.34	81.71	21.49	N/A	N/A	N/A	N/A

Red font = value is higher than 1 standard deviation of healthy control mean.
Blue font = value is lower than 1 standard deviation of healthy control mean.
++N/A = data not available. Patients # 2,5,7,12-14 did not receive PBD; patient #14 did not receive rectal bisacodyl. HRV was not recorded during the baseline of patient #5. Exact time matching of HRV and HRCM recording was not available for motor pattern HRV analysis in patient #5.
Abbreviations:
RSA = respiratory sinus arrhythmia, an indicator of parasympathetic activity
RMSSD = root mean square of successive differences between heartbeats, an indicator of parasympathetic activity
SI = sympathetic index, the Baevsky index as an indicator of sympathetic activity
SI/RSA = autonomic balance
HR = heart rate
HAPW = high amplitude pressure waves

Supplementary Table S4. Predictive capability of HRV parameters for the absence of reflexes in patients with chronic constipation (N=14)

		Sensitivity	Specificity	Likelihood ratio	Sensitivity	Specificity	Likelihood ratio	Sensitivity	Specificity	Likelihood ratio	Sensitivity	Specificity	Likelihood ratio
		Coloanal dyssynergia			Absence of gastrocolic reflex			Absence of vagosacral reflex			Absence of sacral autonomic reflex		
High SI	Supine	0.6	0.43	1.05	0.5	0.5	1	0.50	0.75	2	0.50	0.60	1.25
	Standing	0.8	0.57	1.87	0.83	0.5	1.67	0.75	0.50	1.5	0.62	0.40	1.04
	Baseline	0.2	0.67	0.6	0.6	0.88	5	0.25	0.75	1	0.12	0.50	0.25
	PBD	0.33	0.75	1.33	0.2	0.67	0.61	0.25	0.75	1	0.17	0.50	0.34
	Meal	0.8	0.57	1.87	0.83	0.62	2.22	0.25	0.50	0.5	0.38	0.20	0.48
	Rectal bisacodyl	0.4	0.71	1.40	0.4	0.62	1.07	0.25	0.75	1	0.25	0.40	0.42
Low RSA	Supine	0	1	-	0	1	-	0	1	-	0	1	-
	Standing	0.40	0.86	2.86	0.33	0.88	2.67	0	0.8	0	0.12	0.6	0.31
	Baseline	0	0.86	0	0.2	1	-	0	1	-	0	0.75	0
	PBD	0.67	0.75	2.68	0.67	0.8	3.33	0.25	0.5	0.5	0.17	0	0.17
	Meal	1	0.71	3.50	0.5	0.5	1	0.25	0.5	0.5	0.38	0.20	0.48
	Rectal bisacodyl	1	0.57	2.33	0.6	0.25	0.8	0.5	0	0.5	0.62	0.2	0.78
Low RMSSD	Supine	0	1	-	0	1	-	0	1	-	0	1	-
	Standing	0.2	1	-	0.17	1	-	0	0.75	0	0	0.8	0
	Baseline	0.2	0.83	1.2	0.25	0.86	1.79	0	1	-	0	0.5	0
	PBD	0	0.75	0	0.33	0.8	1.65	0.25	0.75	1	0.17	0.5	0.33
	Meal	0.8	0.83	4.8	0.6	0.57	1.40	0.25	0.5	0.5	0.38	0.25	0.5
	Rectal bisacodyl	1	0.67	3	0.4	0	0.4	0.5	0.25	0.67	0.5	0	0.5
High SI/RSA	Baseline	0.2	0.67	0.6	0.66	0.86	4.67	0.5	0.75	2	0.25	0.5	0.5
	PBD	0.33	0.75	1.32	0.2	0.33	0.3	0.5	0.75	2	0.5	0.67	1.5
	Meal	1	0.57	2.33	0.83	0.5	1.67	0.25	0.5	0.5	0.8	0.5	1.6
	Rectal bisacodyl	1	0.28	1.4	0.6	0.33	0.9	0.25	0	0.25	0.63	0	0.63

Red indicates likelihood ratios greater than 2, suggesting more likelihood of finding an absence of the reflex [62]. The likelihood is higher with higher ratios.

Supplementary Table S5. Autonomic nervous system activity in association with HAPWs in patients with chronic constipation

	Before Mean ± SEM	During Mean ± SEM	After Mean ± SEM	p-value (B-D)	p-value (D-A)
HRV parameters associated with all HAPWs in patients N=9 n=42					
RSA [ln(ms^2)]	5.50 ± 0.52	5.78 ± 0.46	5.63 ± 0.49	0.3607	0.7350
RMSSD (ms)	37.16 ± 11.61	36.58 ± 11.80	37.26 ± 13.20	>0.9999	>0.9999
SI (s^{-2})	144.59 ± 40.73	102.43 ± 24.95	132.54 ± 40.32	0.1187	0.1979
HRV parameters associated with HAPWs in response to PBD N=5 n=6					
RSA [ln(ms^2)]	5.19 ± 0.55	5.32 ± 0.50	4.89 ± 0.64	>0.9999	0.2756
RMSSD (ms)	24.26 ± 5.456	22.48 ± 3.88	20.49 ± 3.86	>0.9999	0.8806
SI (s^{-2})	179.00 ± 83.16	146.34 ± 67.19	215.11 ± 128.06	>0.9999	>0.9999
HRV parameters associated with HAPWs in response to meal N=6 n=12					
RSA [ln(ms^2)]	6.17 ± 0.65	6.43 ± 0.55	6.22 ± 0.63	0.6246	0.2247
RMSSD (ms)	52.10 ± 19.31	53.50 ± 22.68	53.93 ± 21.67	>0.9999	>0.9999
SI (s^{-2})	105.53 ± 35.61	68.75 ± 22.53	145.00 ± 44.83	0.2234	0.0728
HRV parameters associated with HAPWs in response to rectal bisacodyl N=7 n=14					
RSA [ln(ms^2)]	4.81 ± 0.52	5.31 ± 0.50	5.42 ± 0.45	0.1474	>0.9999
RMSSD (ms)	20.00 ± 7.58	23.48 ± 7.98	24.50 ± 9.79	0.5701	>0.9999
SI (s^{-2})	191.69 ± 72.20	107.13 ± 22.56	110.80± 29.88	0.2176	0.8454

N = number of subjects, n= number of HAPWs and HAPW clusters. P-value is determined using ANOVA followed by Bonferroni Multiple Comparison test or Friedman test followed by Dunn's Multiple Comparison test after normality analysis using Shapiro-Wilk Normality test.

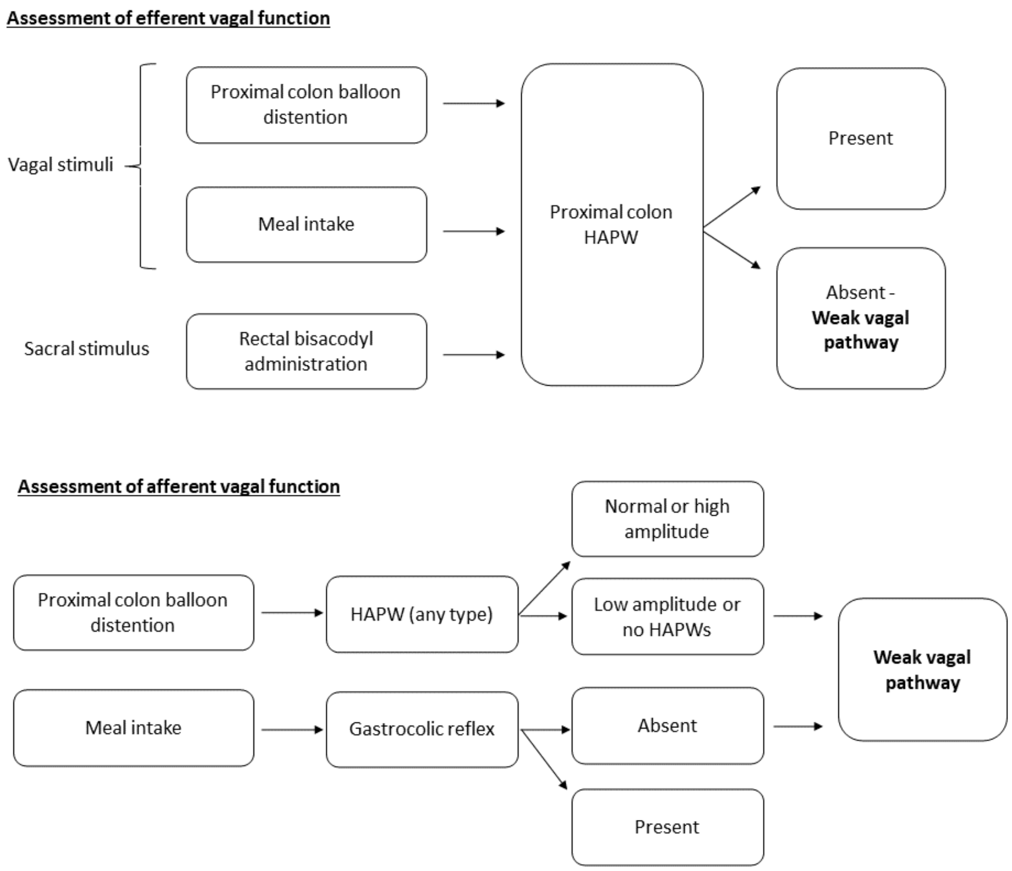

Supplementary Figure S1. Method of assessing vagal pathway function through various stimuli during high-resolution colonic manometry.

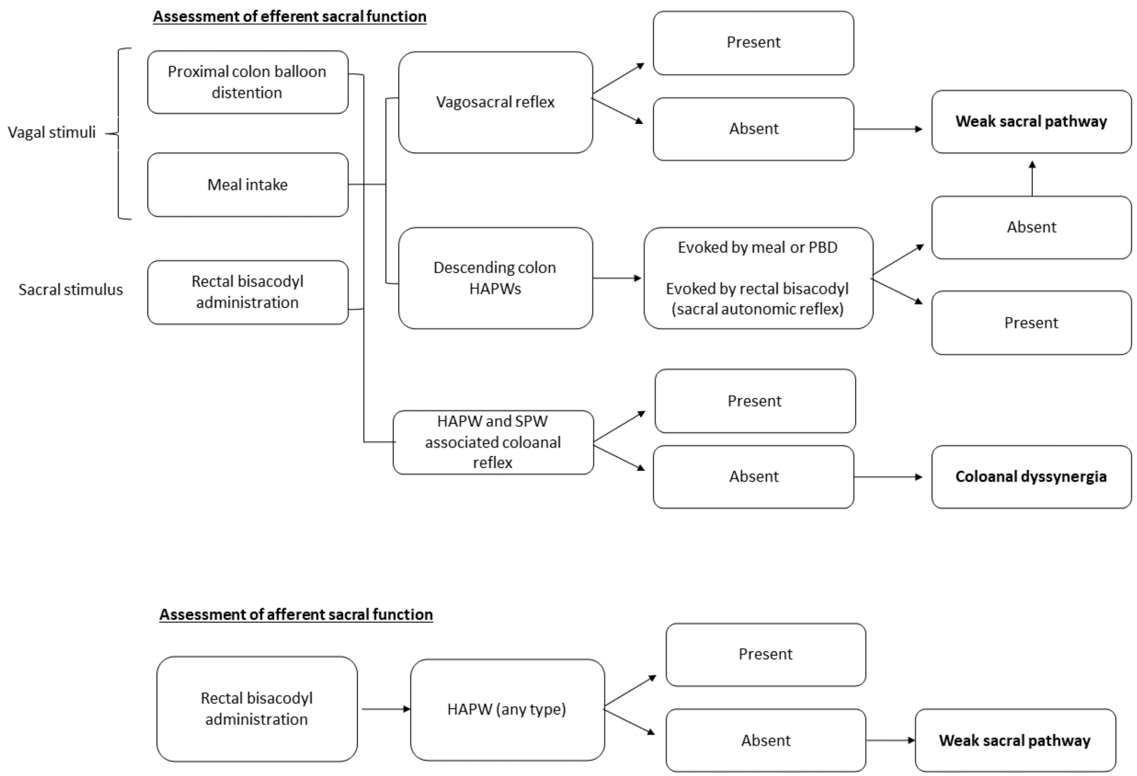

Supplementary Figure S2. Method of assessing sacral pathway function through various stimuli during high-resolution colonic manometry.
